# Integrative evidence construction for resveratrol treatment of nonalcoholic fatty liver disease: preclinical and clinical meta-analyses

**DOI:** 10.3389/fphar.2023.1230783

**Published:** 2023-09-12

**Authors:** Xuan He, Yubing Li, Xinyu Deng, Xiaolin Xiao, Jinhao Zeng

**Affiliations:** ^1^ Department of Pharmacy, Xindu District Shibantan Street Community Healthcare Center, Chengdu, China; ^2^ School of Pharmacy, Chengdu University of Traditional Chinese Medicine, Chengdu, China; ^3^ Department of Gastroenterology, Hospital of Chengdu University of Traditional Chinese Medicine, Chengdu, China

**Keywords:** resveratrol, nonalcoholic fatty liver disease, clinical, preclinical, integrative evidence

## Abstract

**Background:** Resveratrol, a polyphenol found in various plants, is known for its diverse bioactivities and has been explored in relation to nonalcoholic fatty liver disease (NAFLD). However, no high-quality evidence exists regarding its efficacy.

**Objective:** a meta-analysis was conducted to evaluate the potential efficacy of resveratrol in treating nonalcoholic fatty liver disease by analyzing both preclinical studies and clinical trials.

**Method:** PubMed, Embase and Web of Science were searched for the included literature with the criteria for screening. Quantitative synthesis and meta-analyses were performed by STATA 16.0.

**Results:** Twenty-seven studies were included, and the results indicated that resveratrol effectively improved liver function, reduced fatty liver indicators, and affected other indices in preclinical studies. The effective dosage ranged from 50 mg/kg-200 mg/kg, administered over a period of 4–8 weeks. While there were inconsistencies between clinical trials and preclinical research, both study types revealed that resveratrol significantly reduced tumor necrosis factor-α levels, further supporting its protective effect against nonalcoholic fatty liver disease. Additionally, resveratrol alleviated nonalcoholic fatty liver disease primarily via AMPK/Sirt1 and anti-inflammatory signaling pathways.

**Conclusion:** Current meta-analysis could not consistently verify the efficacy of resveratrol in treating nonalcoholic fatty liver disease, but demonstrated the liver-protective effects on nonalcoholic fatty liver disease. The large-sample scale and single region RCTs were further needed to investigate the efficacy.

## Highlights

1. The research firstly constructed the integrative evidence for resveratrol treating NAFLD based on preclinical and clinical meta-analyses.
2. The liver protective effect via AMPK/Sirt1 and anti-inflammatory signals was proved as the key process of resveratrol alleviating NAFLD.
3. Resveratrol did not display the consistent efficacy on NAFLD in both clinical studies and clinical trials.

## 1 Introduction

Nonalcoholic fatty liver disease (NAFLD), characterized by the accumulation of fat in liver cells due to metabolic disorders, is a significant public health concern in the context of improving living standards worldwide. Its prevalence is more severe than other infectious diseases, estimated to be 60%–90% in patients with obesity, 28%–50% in patients with type 2 diabetes, and 17%–33% in the general population (Dietrich and Hellerbrand, 2014). In Asian countries, particularly China, the incidence has rapidly increased over the past 2 decades, with no signs of decline. NAFLD poses a major health risk as approximately 25% of patients may progress to cirrhosis and become susceptible to hepatocellular carcinoma (Powell et al., 2021). Therefore, there is an urgent need for specific drugs to slow down this progression; however, the efficacy of available agents for treating NAFLD remains controversial.

Natural products have long been considered a valuable source for drug development. For example, artemisinin, a classical sesquiterpene lactone, has made significant strides in malaria therapy and saved numerous lives (Ma et al., 2020a). Taxol, initially derived from the Pacific Yew plant in 1963, has shown remarkable efficacy against various malignant tumors (“Clinical pharmacology and metabolism, 1993 of Taxol (paclitaxel): update 1993—PubMed,” n.d.). Apart from these well-known compounds, there are numerous promising natural compounds that warrant further exploration. Resveratrol, also known as 3–4′-5-trihydroxystilbene, is a polyphenol found in various plants such as Vitis vinifera, Cassia tora, and Polygonum cuspidatum. Recent studies have demonstrated its significant effects on reducing triacylglycerol levels and regulating lipid metabolism (Chaplin et al., 2018). Consequently, the hepatoprotective effects of resveratrol in NAFLD have garnered substantial attention in both experimental and clinical settings. However, whether resveratrol exhibits consistent effects on NAFLD *in vivo* and in randomized clinical trials (RCTs), as well as its precise mechanism of action, are crucial questions that require confirmation for further drug development.

Meta-analysis has gained prominence in evidence-based medicine (EBM) in recent years. While conventional meta-analyses primarily rely on RCTs, there has been a growing trend toward conducting preclinical meta-analyses to complement the existing evidence. Preclinical data are easily accessible and can provide more detailed information regarding the effects of specific dosages and durations. Moreover, this approach allows for a comprehensive understanding of underlying mechanisms. Consequently, combining preclinical and clinical studies to construct integrative evidence (Samadi et al., 2022). Firstly, it provides a more robust and convincing body of evidence beyond individual meta-analyses. Secondly, it yields more precise information about the underlying action of therapeutic agents during disease treatment.

While resveratrol holds promise as a candidate for NAFLD treatment based on existing research, high-quality evidence is still needed to confirm its bioactivity and efficacy. To provide further insights into dosage, duration, and the underlying mechanisms for clinical application, it is imperative to construct integrative evidence based on both preclinical and clinical perspectives. Consequently, this study employed a meta-analysis to comprehensively evaluate the efficacy and mechanism of resveratrol in the treatment of NAFLD. The findings will contribute to establishing a scientific foundation for its pharmacological characteristics and potential clinical applications.

## 2 Methods

This research adhered to the Preferred Reporting Items for Systematic Reviews and Meta-Analyses (PRISMA) statements (Moher et al., 2009).

### 2.1 Data sources and literature selection

Potential database were available from PubMed (https://pubmed.ncbi.nlm.nih.gov/), Embase (https://www.embase.com/) and Web of Science (https://www.webofscience.com/). All the search time was from the inception of database to August 2022 with specific search strategies including main PICOS elements such as “nonalcoholic fatty liver disease” and “resveratrol.” The detailed search strategy in PubMed was listed as the representative in Supplementary Table S1.

### 2.2 Eligibility criteria

The inclusion criteria for including preclinical literature were as follows: 1) Population (P) and study (S): rodent models to establish NAFLD; 2) Intervention (I) and comparison (C): the experimental group was only received resveratrol at certain dose, whereas the model group was received equivalent amounts of non-functional substances by the same route; 3) Outcome (O): the primary outcome measures contained histopathology, liver function and lipid metabolism. Other index or parameter relevant to mechanism was selected as secondary outcome measures. The criteria for including clinical trials were as follows: 1) patients diagnosed with NAFLD and without any other complications; 2) patients in experimental group were treated with resveratrol or related supplement, whereas patients in control group were treated with conventional therapy; 3) the outcome measures were closely to clinical efficacy and liver function; 4) type of study belongs to RCT.

Exclusion criteria for preclinical literature were as follows: 1) Population (P) and study (S): Not belonging to NAFLD models or other types of articles such as review, editorial, meta-analysis; 2) Intervention (I): treatment with other prescriptions or combinations with other agents; 3) Comparison (C): comparison with other agents; 4) Outcome (O): no specific outcome index or no available data. Exclusion criteria for clinical trials were as follows: 1) patients with other complications or serious diseases; 2) patients in experimental or control group were treated with other kinds of natural products or related supplements; 3) no clinical outcome measures available; 4) non-RCT study design.

### 2.3 Data extraction

Two researchers independently evaluated and crosschecked all the including articles to refine data according to the following criteria: 1) study ID with first author’s name and the publication year were recorded; 2) characteristics of different types containing RCTs and experiments were extracted based on age, gender (male/female), sample size, model type, species, weight and so on; 3) the regimen features described in intervention and comparison as dosage, frequency or period of administration, delivery route, vehicle; 4) primary and secondary outcomes in RCTs and experiments; 5) mechanisms underlying action of resveratrol against NAFLD. During the experiment, multiple doses of resveratrol were extracted for dosage analysis. Meanwhile, only the information of high dose groups was refined for comparison (Li et al., 2022a). Regarding continuous variables usually displayed as graph, WebPlotDigitizer was applied to extract the mean score and standard deviation (SD) from both intervention and model groups.

### 2.4 Risk of bias and quality of evidence assessment

The SYRCLE list proposed by Hooijmans including 10 items were also used to evaluate preclinical studies (Hooijmans et al., 2014). A risk of bias assessment for clinical studies was performed according to Cochrane collaboration’s tool through the Review Manager 5.4 software. Two researchers independently carried out the progress and if their assessment were inconsistent, a third researcher would discuss and judge the results for consensus.

### 2.5 Quantitative synthesis and statistical analysis

STATA 16.0 software was utilized for all quantitative synthesis and meta-analyses. Dichotomous variables, especially for clinical effectiveness rate, were presented as relative risk (*RR*), while continuous variables, including most indices such as liver function and lipid metabolism, were displayed as standardized mean difference (S*MD*). In addition, dichotomous and continuous were both with 95% confidence intervals (*CI*) calculated. The heterogeneity was mainly evaluated according to I-square (*I* ^*2*^) index with a different meaning to concern highly heterogeneous data (*I* ^*2*^ > 50%) for randomized-effects model and homogeneous data (*I* ^*2*^ ≤ 50%) for fixed-effects model. Sensitivity analysis was also use for exploring the possible sources of heterogeneity. In addition, publication bias was determined using the Egger’s test with |t| < 0.05 considered as potential publication bias. Specifically, dose-duration interval analysis was applied to describe the optimal treatment range in preclinical meta-analysis. Mechanism in preclinical studies were also summarized.

## 3 Results

### 3.1 Research screening

**FIGURE 1:**
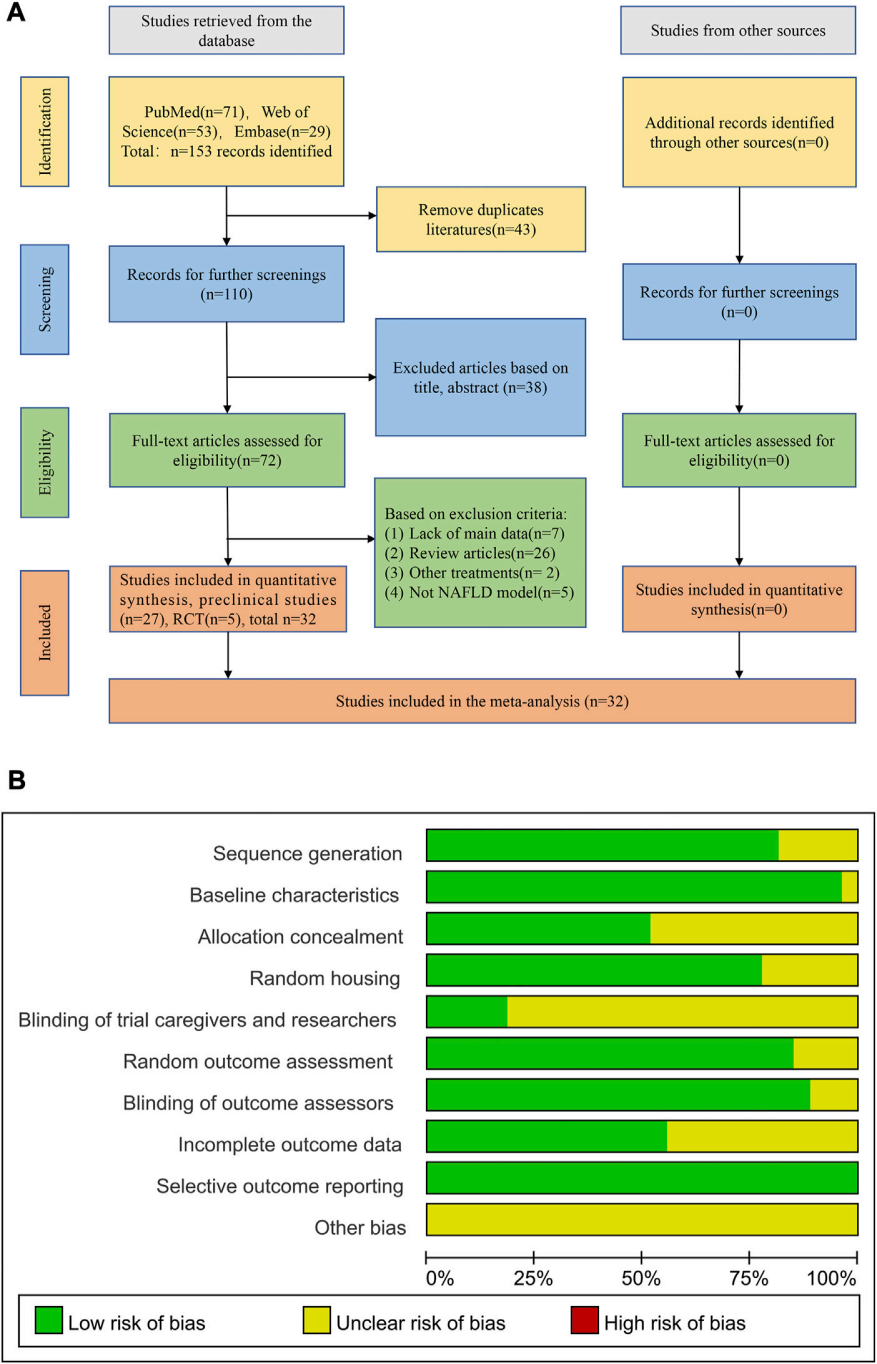
Flowchart and the quality assessment of included studies. **(A)** Flowchart of selection process; **(B)** Quality assessment of preclinical studies.

A total of 153 literatures were obtained through preliminary retrieval including 71 records from PubMed, 53 records from Web of Science and 29 records from Embase. After removal of duplicated articles, 110 articles were left for further screening. Thirty-eight articles were excluded based on the title and abstract reading. Then, the remaining 72 full-text articles were obtained for further screening. In these articles, 7 records lacked of main data, 26 records were review article, 2 records combined other treatment besides resveratrol and 5 records with no NAFLD model during investigation. Ultimately, a total of 27 preclinical studies (Gómez-Zorita et al., 2012; Poulsen et al., 2012; Andrade et al., 2014; Li et al., 2014; Yang and Lim, 2014; Ji et al., 2015; Ali et al., 2016; Kessoku et al., 2016; Tian et al., 2016; Ding et al., 2017; Guo et al., 2017; Hajighasem et al., 2018; Cheng et al., 2019; Hajighasem et al., 2019; Teng et al., 2019; Xu et al., 2019; Chang et al., 2020; Che et al., 2020; Huang et al., 2020; Wang et al., 2020; Du et al., 2021; Hu et al., 2021; Milton-laskibar et al., 2021; BinMowyna et al., 2022; Li et al., 2022b; Yuan et al., 2022; Zhao et al., 2022) and 5 RCT (Chachay et al., 2014; Faghihzadeh et al., 2014; Chen et al., 2015; Heebøll et al., 2016; Asghari et al., 2018) literatures met the inclusion criteria for further analyses (Figure 1A).

### 3.2 Characteristics of included studies

The analysis included 27 preclinical studies, involving a total of 480 animals, with 241 animals in the experimental group and 239 animals in the model group. The animal species used in the studies were Sprague–Dawley rats, C57BL/6 mice and Wistar rats. The animal models included a high-fat diet and a methionine-choline-deficient diet (MCD), with two studies using Obese Zucker rats as the pathological model. The duration of the administration was from 4 weeks to 20 weeks, and the dosage was from 20 mg/kg to 400 mg/kg. The animals in experimental group were all treated with resveratrol based on NAFLD model. The main outcome indicators included liver function indexes such as ALT, AST and fat indexes such as TG, TC, HDL-C, LDL-C. Some other pharmacological indexes such as serum insulin, body weight, HOMA-IR were as recorded. In addition, the indicators related to oxidative stress and inflammation containing ROS, MDA, GSH, IL-6, IL-1β and TNF-α were also included (Supplementary Table S2).

The analysis also included 5 RCTs with a total of 216 patients diagnosed with NAFLD, divided equally between the experimental and control groups (108 patients in each group). Patients in the experimental group were treated with resveratrol, and those in the control group received a placebo. The age of the participants in the included trials ranged from 39 to 48 years. The duration of administration ranged from 8 to 24 weeks, with a follow-up period of 8–25 weeks. The dosage of resveratrol administered in the trials ranged from 300 to 3,000 mg/kg. The primary outcome indicators included body obesity, liver function, and fat indices. Additionally, we also included indicators related to insulin resistance and inflammation such as serum insulin, glucose, and TNF-α (Supplementary Table S3).

### 3.3 Quality evaluation

The methodological quality was assessed by the modified SRYCLE 10-item checklist with the average score of 6.56 points in preclinical studies (Supplementary Figure S1). Twenty two researches adapt the random allocation method to separate the animals during grouping. All these studies have been rigorously peer reviewed and 26 studies reported the animal were at similar baseline. Allocation concealment was declared in 13 studies and 21 studies described that the animals of different groups were randomly placed in the same environment. Only 5 studies performed blinding during the research. Twenty four studies reported that the animals were randomly selected in the evaluation results and the outcome assessors were blinded. All studies were free of selective results reporting (Figure 1B) and the risk bias summary of 27preclinical studies (Supplementary Figure S1).

The RCT studies were assessed using seven aspects of methodology. The overall quality of the studies showed a relatively low risk of bias, with an average score of 5 out of 7 points. All studies reported on selection bias and performance bias. Three studies reported allocation concealment, and two studies described blinding of outcome assessment. Furthermore, all studies reported incomplete outcome data and other biases. However, no selective reporting bias was observed in any of the included studies (Supplementary Figure S2).

### 3.4 Effect of resveratrol in preclinical studies

#### 3.4.1 The effect on liver function

**FIGURE 2:**
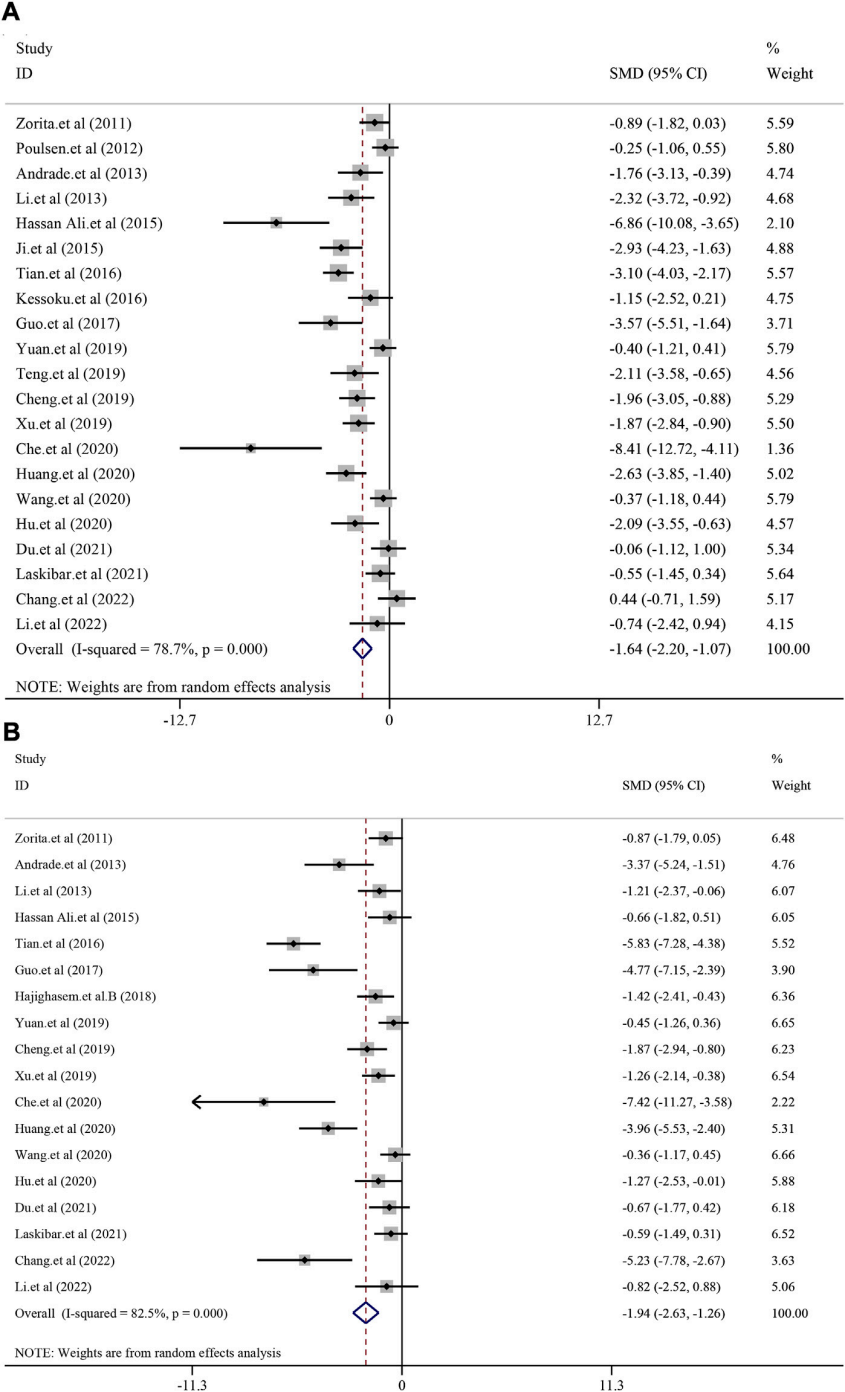
Effect of resveratrol on ALT and AST level in preclinical studies. **(A)** Pooled effect of ALT; **(B)** Pooled effect of AST.

According to the included studies, a total of 21 studies with 362 animals were included in this analysis and random-effect model was used to combined effect size (*I* ^*2*^ = 78.7%, *p* < 0.0001). The results showed that treatment with resveratrol could significantly reduce the ALT level in liver function [*SMD* = −1.64, 95%*CI* (−2.20, −1.07), *p* < 0.0001] (Figure 2A). Eighteen studies with 316 animals showed the level of AST after treatment for resveratrol. The heterogeneity analysis suggested that there was significant heterogeneity (*I* ^2^ = 82.5%, *p* < 0.0001) and the random-effect model was used. The meta-analysis demonstrated there was significant reduction effect in resveratrol group compared with model group [*SMD* = −1.94, 95*%CI* (−2.63,-1.26), *p* < 0.0001] (Figure 2B).

#### 3.4.2 The effect on fat liver indicators

**FIGURE 3:**
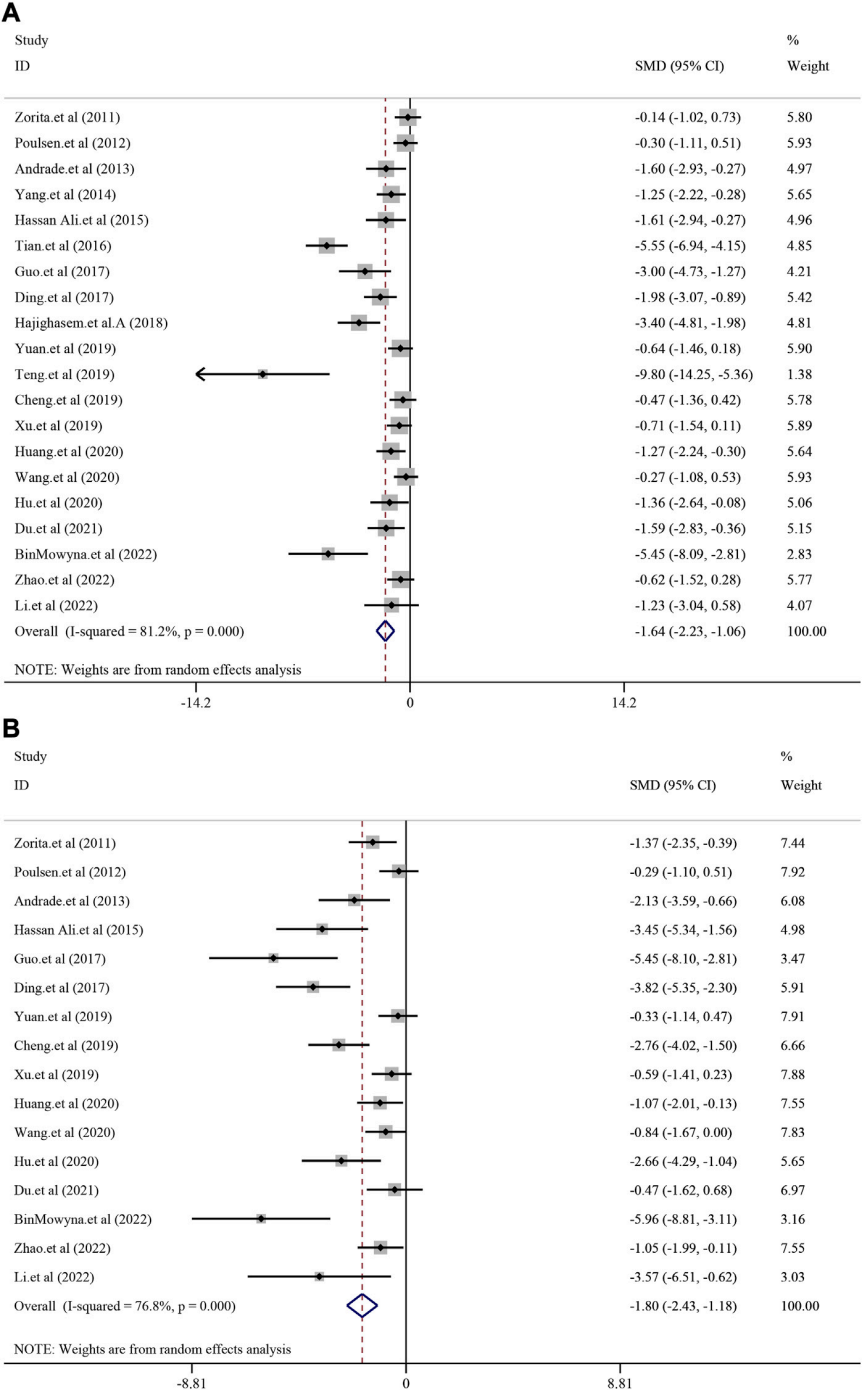
Effect of resveratrol on TG and TC level in preclinical studies. **(A)** Pooled effect of TG; **(B)** Pooled effect of TC.

A total of 20 studies with 368 animals reported the TG level after treatment for resveratrol. Since the result revealed a statistically significant heterogeneity (*I* ^2^ = 81.2%, *p* < 0.0001), the random-effect model was used. Meta-analysis demonstrated that there was significant reduction in resveratrol group compared with model group [*SMD* = −1.64, 95*%CI* (−2.23,−1.06), *p* < 0.0001] (Figure 3A). TC level was reported in 16 studies with 274 animals. Random-effect model was used due to high heterogeneity (*I* ^2^ = 76.8%, *p* < 0.0001) and resveratrol demonstrated remarkable decrease in TC [*SMD* = −1.80, 95%*CI* (−2.43, −1.18), *p* < 0.0001] (Figure 3B). Meanwhile, 13 studies with 208 animals assessed LDL-C level. The random-effect model was used to make further meta-analysis due to a statistically significant heterogeneity (*I* ^2^ = 77.4%, *p* < 0.0001). Compared with model group, the resveratrol group manifested a significant reducing effect on LDL-C level [*SMD* = −2.01, 95%*CI* (−2.77, −1.25), *p* < 0.0001] (Supplementary Figure S3AA). Similarly, 12 studies with 210 animals revealed the HDL-C level. The random-effect model was used due to high heterogeneity (*I* ^2^ = 85.6%, *p* < 0.0001). The meta-analysis demonstrated that there was increasing trend of HDL-C in resveratrol group compared with model group, but there was no significance [*SMD* = 0.71, 95*%CI* (−0.11,1.54), *p* = 0.091] (Supplementary Figure S3BB). As for the index of serum insulin (*I* ^2^ = 90.1%, *p* < 0.0001), resveratrol could remarkably reduce the level compared with treatment in model group [*SMD* = −2.15, 95*%CI* (−3.54,-0.75), *p* = 0.003] (Supplementary Figure S4).

#### 3.4.3 The effect on body weight and HOMA-IR

Thirteen studied with 256 animals reported body weight after treatment for resveratrol. Due to the significant heterogeneity (*I* ^*2*^ = 86.8%, *p* < 0.0001), the random-effect model was used. Resveratrol group manifested significant reduction on body weight compared with treatment in model group [*SMD* = −1.27, 95%*CI* (−2.09, −0.45), *p* = 0.002] (Supplementary Figure S5A). As for HOMA-IR level, 5 animal experiments demonstrated relatively low heterogeneity (*I* ^*2*^ = 20.5%, *p* = 0.284). Thus, the result based on fixed model revealed that resveratrol could remarkably decrease HOMA-IR [*SMD* = −1.74, 95%*CI* (−2.24, −1.24), *p* < 0.0001] (Supplementary Figure S5B).

#### 3.4.4 The effect on oxidative stress indexes

In order to investigated the effect of resveratrol on anti-oxidation in NAFLD model, MDA, SOD and GSH was analyzed. There were 13, 11 and 7 studies reporting MDA, SOD and GSH respectively. In addition, random-effect model was used due to significant heterogeneity (*I* ^*2*^ = 74.8%, *I* ^2^ = 75.6%, *I* ^2^ = 84.0%, *p* < 0.0001). The results demonstrated that treatment with resveratrol could significantly reduce the MDA level compared with treatment in model group [*SMD* = −2.22, 95%*CI* (−2.95, −1.50), *p* < 0.0001] (Supplementary Figure S6A). Similar result also reflected in SOD [*SMD* = 1.92, 95*%CI* (1.18,2.65), *p* < 0.0001] (Supplementary Figure S6B) and GSH [*SMD* = 1.74, 95%*CI* (0.64, 2.85), *p* = 0.002] (Supplementary Figure S7).

#### 3.4.5 The effect on inflammatory indexes

Several indexes relevant to anti-inflammatory effect including TNF-α, IL-6 and IL-1β were investigated. There were 17, 13 and 8 studies reporting TNF-α, IL-6 and IL-1β respectively. Random-effect model was used due to significant heterogeneity (*I* ^*2*^ = 83.9%, *I* ^2^ = 85.1%, *I* ^2^ = 60.8%, *p* < 0.0001, *p* = 0.013). The results demonstrated that treatment with resveratrol could significantly reduce the TNF-α level compared with treatment in model group [*SMD* = −2.62, 95%*CI* (−3.46, −1.78), *p* < 0.0001] (Supplementary Figure S8A). Similar result also reflected in IL-6 [*SMD* = −3.14, 95%*CI* (−4.23, −2.05), *p* < 0.0001] (Supplementary Figure S8B) and IL-1β[*SMD* = −2.10, 95%*CI* (−2.87, −1.33), *p* < 0.0001] (Supplementary Figure S9).

### 3.5 Subgroup analysis of preclinical studies

#### 3.5.1 Subgroup analysis of TG

The representative indices of NAFLD, including TG and ALT were analyzed via subgroup based on animal species and resveratrol dosage. Nineteen studies have reported the TG level after resveratrol treatment for NAFLD. Based on the subgroups of animal species, the results manifested that resveratrol could reduce the level of blood TG in the Mice species [*n* = 212, *SMD* = −1.77, 95*%CI*(−2.60, −0.93), *p* < 0.0001]. As for Rats species, resveratrol also demonstrated significant effect on blood TG level [*n* = 132, *SMD* = −1.68, 95*%CI*(-2.70, −0.66), *p* = 0.001] (Supplementary Figure S10). In the subgroup for resveratrol dosage, we defined the dosage of resveratrol was less than 50 mg/kg as the low dose, 50–100 mg/kg as the medium dose, and more than 100 mg/kg as the high dose. The result manifested that resveratrol at low dose could reduce the blood TG level [*n* = 148, *SMD* = −1.91, 95*%CI*(−3.20, −0.63), *p =* 0.004]. Middle and high dosage of resveratrol could also reduce the blood TG respectively [*n* = 118, *SMD* = −0.91, 95*%CI*(−1.30, −0.52), *p <* 0.001; *n* = 80, *SMD* = −3.3, 95*%CI*(-5.41, −1.25), *p* = 0.002] (Supplementary Figure S11). From this subgroup analysis we would speculate that resveratrol displayed continued and extensive effect on reducing TG in NAFLD.

#### 3.5.2 Subgroup analysis of ALT

Twenty one studies have reported the serum ALT level after resveratrol treatment for NAFLD. According to the subgroup of animal species, the results manifested that resveratrol could reduce the level of ALT in Mice species [*n* = 242, *SMD* = −1.88, 95%*CI*(-2.69, −1.08), *p* < 0.0001]. The subgroup analysis for Rat species also demonstrated similar effect [n = 120, *SMD* = −1.44, 95%*CI*(−2.30, −0.58), *p* = 0.001] (Supplementary Figure S12). In the resveratrol dosage subgroup, the results showed that resveratrol at a low dose could reduce serum ALT level [*n* = 142, *SMD* = −2.32, 95*%CI*(−3.49, −1.14), *p <* 0.001]. Middle and high dosage of resveratrol could also reduce the serum ALT respectively [*n* = 128, *SMD* = −1.17, 95*%CI*(-1.93, −0.40), *p =* 0.003; *n* = 68, *SMD* = −2.13, 95*%CI*(-3.70, −0.57), *p* = 0.008] (Supplementary Figure S13). Apart from TG level, resveratrol also displayed continued and extensive effect on reducing ALT in NAFLD.

### 3.6 Efficacy of resveratrol in clinical trials

#### 3.6.1 Efficacy of resveratrol on anthropometric indices

Five included RCTs were used to assess the anthropometric indices, including body weight (BW), body mass index (BMI), waist circumference (WC), and waist-to-hip ratio (WHR). No significant heterogeneity was found in BW (*I* ^*2*^ = 0.0%, *p* = 0.956), BMI (*I* ^*2*^ = 0.0%, *p* = 0.823) and WHR (*I* ^*2*^ = 0.0%, *p* = 0.730), except for WC (*I* ^*2*^ = 93.6%, *p* < 0.0001). The result indicated that there was just the trend of decrease but no significance after treatment with resveratrol compared with placebo in BW [*SMD* = − 0.08, 95%*CI* (−0.35, 0.18), *p* = 0.544], BMI [*SMD* = − 0.07, 95%*CI* (−0.33, 0.20), *p* = 0.633], WC [*SMD* = −0.73, 95%*CI* (−1.97, 0.51), *p* = 0.249] and WHR [*SMD* = −0.12, 95%*CI* (−0.40, 0.16), *p* = 0.405] (Supplementary Figures S14, 15).

#### 3.6.2 The efficacy on liver function

**FIGURE 4:**
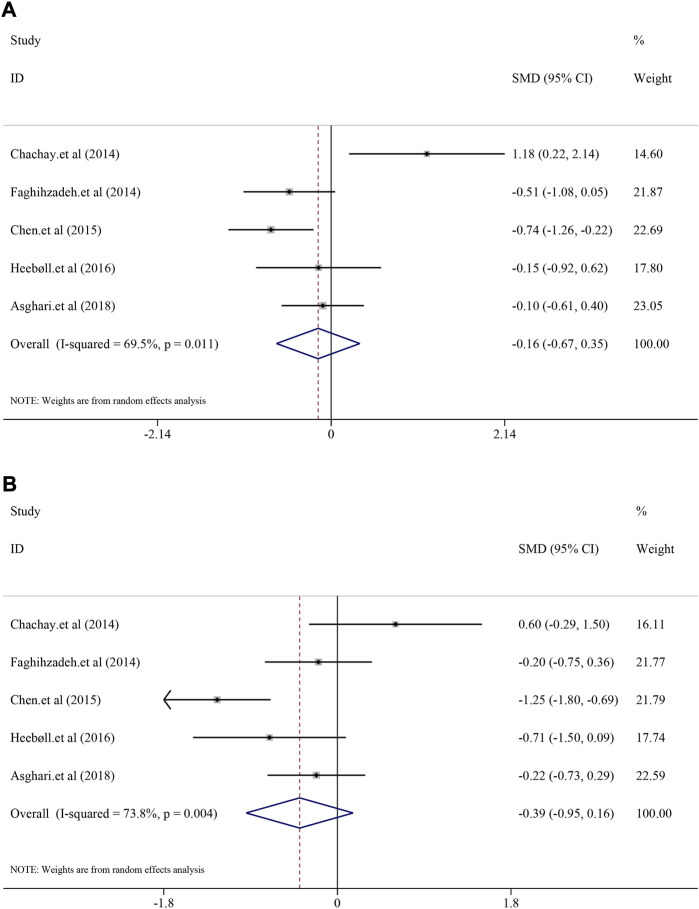
Efficacy of resveratrol on ALT and AST level in clinical studies. **(A)** Pooled efficacy of ALT; **(B)** Pooled efficacy of AST.

As for liver function indexes such as ALT and AST, random-effect model was used for analyses due to high heterogeneity (*I* ^*2*^ = 69.5%, *p* = 0.011; *I* ^*2*^ = 73.8%, *p* = 0.004). Interestingly, the result suggested that there was no significant efficacy of resveratrol on ALT level in NAFLD patients [*SMD* = −0.16, 95%*CI* (−0.67, 0.35), *p* = 0.542] (Figure 4A), nor was the efficacy in AST [*SMD* = −0.39, 95%*CI* (−0.95, 0.16), *p* = 0.167] (Figure 4B).

#### 3.6.3 The efficacy on fat liver indicators

**FIGURE 5:**
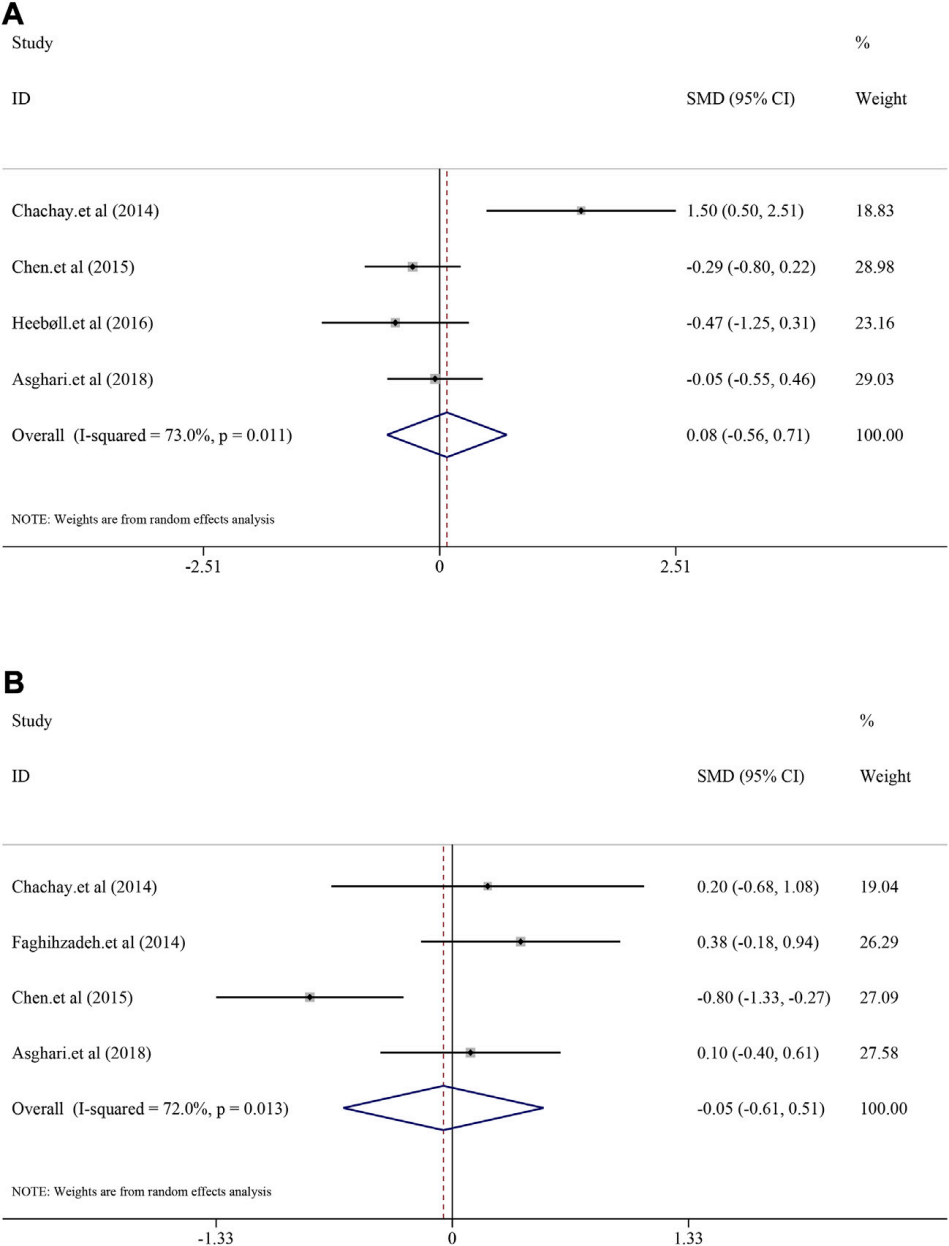
Efficacy of resveratrol on TG and TC level in clinical studies. **(A)** Pooled efficacy of TG; **(B)** Pooled efficacy of TC.

Comparison of fat liver indicators containing TG, TC, LDL and HDL between the resveratrol and control groups were also analyzed. The heterogeneity of TG, TC and LDL was generally high (*I* ^*2*^ = 73.0%, *p* = 0.011; *I* ^*2*^ = 72.0%, *p* = 0.013; *I* ^*2*^ = 81.6%, *p* < 0.0001), whereas the heterogeneity of HDL was minor (*I* ^*2*^ = 0.0%, *p* = 0.891). Interestingly, resveratrol did not demonstrate significant decrease in TG [*SMD* = 0.08, 95%*CI* (−0.56, 0.71), *p* = 0.813] and TC [*SMD* = −0.05, 95%*CI* (−0.61, 0.51), *p* = 0.862] (Figure 5). Similar mild regulated influence was also reflected in LDL [*SMD* = 0.13, 95%*CI* (−0.53, 0.79), *p* = 0.694] and HDL [*SMD* = 0.12, 95%*CI* (−0.14, 0.39), *p* = 0.362] (Supplementary Figure S16).

#### 3.6.4 The efficacy on other indicators

Insulin, glucose and HOMA-IR and TNF-α were also evaluated. Unfortunately, resveratrol did not efficiently regulate insulin [(*I* ^*2*^ = 50.9%, *p* = 0.106), *SMD* = 0.09, 95%*CI* (−0.38, 0.56), *p* = 0.709], HOMA-IR [(*I* ^*2*^ = 56.7%, *p* = 0.074), *SMD* = −0.05, 95%*CI* (−0.55, 0.46), *p* = 0.857] and glucose [(*I* ^*2*^ = 28.3%, *p* = 0.242), *SMD* = −0.58, 95%*CI* (−0.90, −0.26), *p* < 0.0001] (Supplementary Figure S17, 18). From the result could be seen that It could specifically downregulate TNF-α level [(*I* ^*2*^ = 0.0%, *p* = 0.518), *SMD* = −0.45, 95%*CI* (−0.77, −0.13), *p* = 0.006] (Supplementary Figure S19).

### 3.7 Comparison of dosage and duration ranges in preclinical studies and clinical trials

**FIGURE 6:**
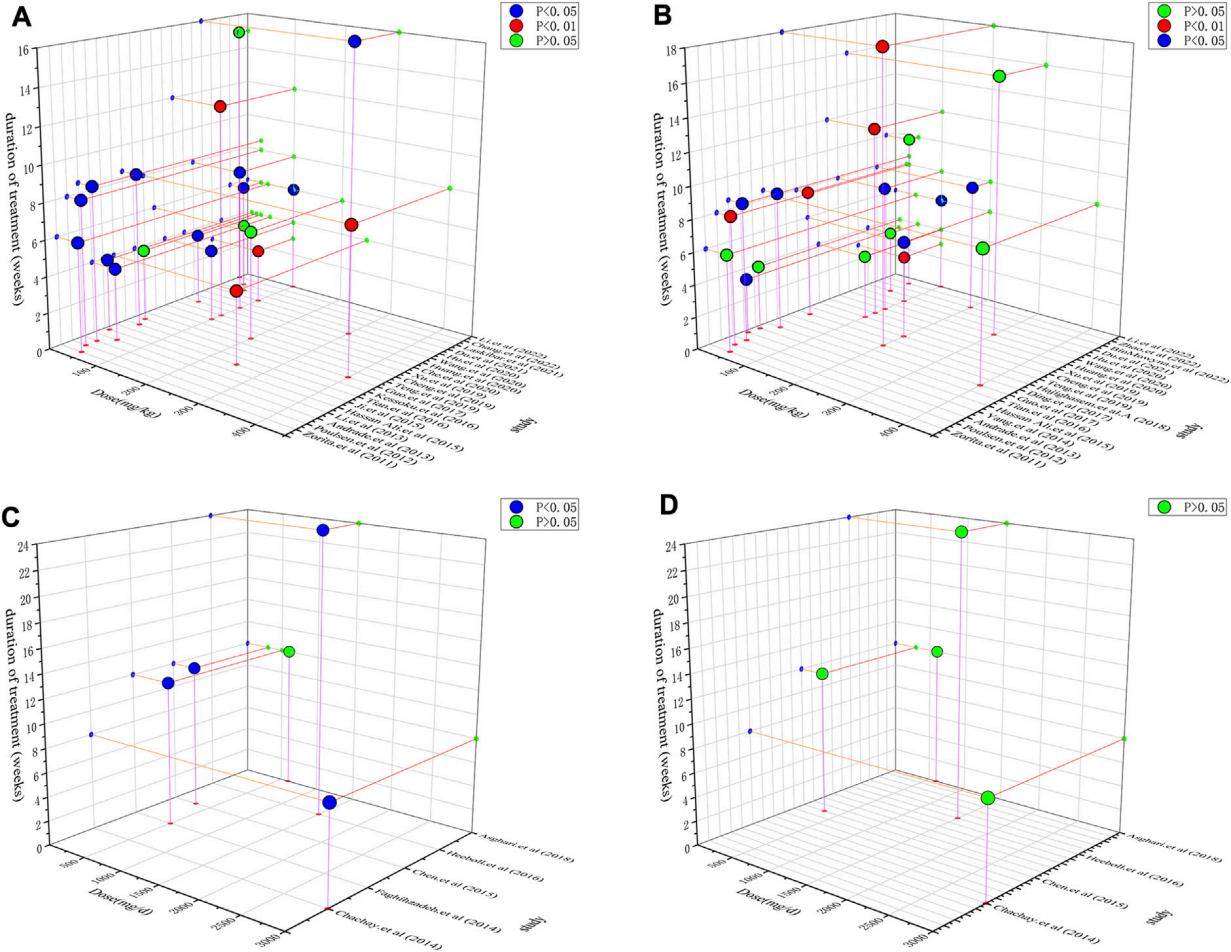
Dose/duration-effect relationship in preclinical studies and clinical trials. **(A)** ALT in preclinical studies. **(B)** TG in preclinical studies. **(C)** ALT in clinical trials. **(D)** TG in clinical trials.

The inconsistent results between preclinical studies and clinical trials are somewhat confusing. Because dosage and duration were the factors that influenced efficacy, a dose/duration–effect relationship plot was constructed. Resveratrol at doses of 50–200 mg/kg and a time range of 4–8 weeks exhibited a remarkable alleviation effect on ALT and TG (Figures 6A,B). Moreover, in preclinical studies, resveratrol appeared to regulate ALT more significantly than TG. This trend was also observed in clinical trials. Figures 6C,D demonstrate that resveratrol more effectively alleviates ALT than TG. Furthermore, four trials reported the efficacy of resveratrol on ALT, whereas only one trial did not observe efficacy. In contrast, the abovementioned four trials reported that resveratrol could not downregulate TG levels. The general dosage ranged from 500 to 3,000 mg/day and duration was 8–24 weeks. After dosage conversion, no significant difference was observed between preclinical studies and clinical trials. This inconsistency may be due to the small sample size of the current clinical RCTs. But as far as the current experimental evidence is concerned, resveratrol can show a better downregulation trend on ALT, TG in preclinical and clinical trials. It suggests that resveratrol exerts both hepatoprotective and lipid metabolism regulating effects.

### 3.8 Publication bias

To determine the possibility of publication bias, Egger’s test was used in preclinical studies. ALT and TG were selected as the main analysis subjects. The outcome index for ALT was *t* = −3.72; and the absolute value was >0.05. On the other hand, the TG outcome index was *t* = −5.36; and the absolute value was also greater than 0.05. Taken together, these findings indicate that there is no obvious publication bias for ALT and TG (Supplementary Figure S20).

### 3.9 Possible molecular mechanism of resveratrol on NAFLD based on preclinical studies

**FIGURE 7:**
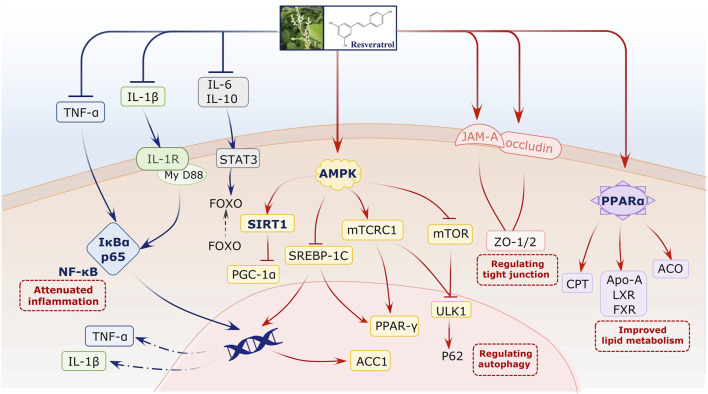
Possible molecular mechanism of resveratrol on NAFLD.

We elucidated the possible molecular mechanism of resveratrol on NAFLD based on preclinical studies. For this, network regulation was conducted using multiple bioactive targets. Many studies have reported that resveratrol can attenuate the adenosine 5′-monophosphate (AMP)-activated protein kinase (AMPK)/sirtuin 1 (Sirt1) signaling pathway. This pathway plays a vital role during NAFLD progress. It not only regulates lipid metabolism but also indirectly affects cell death and autophagy. Second, resveratrol can reverse inflammation-related signals. It primarily downregulates IL-6, IL-1β, and TNF-α, with the NF-κB signaling pathway being the core pathway. In addition, studies have reported that the PPAR-α signal for lipid metabolism improvement is involved during this regulated process. Furthermore, tight junctions, to prevent gut dysbiosis, are associated with the bioactivity of resveratrol on NAFLD (Figure 7, Supplementary Table S4).

## 4. Discussion

### 4.1 Outcome profile and originality

Clarifying the efficacy and mechanism of agents on diseases during drug development is extremely crucial. However, these two issues are generally limited to the focus on a specific aspect in single research. Therefore, meta-analysis provides a foundation for combining multidimensional results to collect robust evidence for these two issues. In the present study, we attempted to collect mixed data to confirm the role of resveratrol in NAFLD. Using preclinical studies and clinical trials, our study is the first to develop an integrated approach for evaluating efficacy and in-depth mechanism.

Interestingly, our findings suggest that resveratrol can not only reverse worse liver function and NAFLD-induced lipid metabolism but also comprehensively regulate the inflammatory and apoptotic status of the hepatocyte microenvironment based on preclinical studies. However, several inconsistencies were observed in clinical trials. The meta-analysis suggests that resveratrol exhibits the trend of improving liver function but does not exhibit efficacy on fatty liver indicators and other indices. Furthermore, a consistent result was observed for TNF-α regulation. These findings indicate that resveratrol exhibits liver-protective efficacy by alleviating inflammation. Moreover, preclinical studies have suggested that resveratrol regulates NALFD in a network-dependent manner. Important signals, including AMPK/Sirt1, NF-κB, and PPAR-α, are involved in this process. Tight junctions relevant to the gut are also regulated. Our study provides comprehensive evidence for the mechanism of resveratrol on NAFLD. Taken together, both preclinical studies and clinical trials have demonstrated the liver-protective efficacy of resveratrol to some extent. However, its regulatory effects on fatty liver indices may not be obvious and remarkable.

### 4.2 Potential reasons for clarifying the inconformity of preclinical and clinical studies

Based on clinical trials, we unexpectedly observed the partially invalid efficacy of resveratrol on NAFLD. In other words, there is inconformity between preclinical and clinical studies. We hypothesize three possible reasons for this result. First, the RCTs have a relatively small sample size, possibly affecting efficacy. The samples in each arm included five trials, with 10–30 patients. The small sample size of these RCTs may increase the risk of false-negative results and therefore interfere with the real efficacy. Second, the clinical trials were conducted in different regions with multiple ethnicities. For example, European or Asian individuals have different dietary habits and physique, and therefore, different degrees of NAFLD. It is difficult to eliminate these confounding factors in meta-analysis, invalidating the entire analysis. Third, resveratrol may exhibit a liver-protective effect rather than a fatty liver-alleviating effect. In particular, many studies have reported that resveratrol can be an effective agent for various liver diseases (Ma et al., 2020b; Lu et al., 2021; Liang et al., 2022). However, this effect is mainly relevant to anti-inflammatory characteristics. This could explain why resveratrol effectively downregulated TNF-α and alleviated liver function indices in both two kinds of studies.

### 4.3 Mechanism by which resveratrol treats NAFLD

The evidence comprehensively verified that resveratrol can alleviate NAFLD via multiple signaling pathways. The AMPK/Sirt1 pathway, commonly mentioned in the included studies, plays a critical role in NAFLD progression. In particular, the core signal Sirt1 is a metabolic regulator that controls lipid metabolism, including fatty acid synthesis, oxidation, and adipogenesis (Ezhilarasan and Lakshmi, 2022; Zeng and Chen, 2022). Therefore, the activation of the AMPK/Sirt1 pathway by resveratrol can essentially improve hepatocyte microenvironment and fat accumulation (Zhou et al., 2021). Neither preclinical studies nor clinical trials confirmed the significant downregulation of TNF-α by resveratrol. Further investigation of the mechanism revealed the involvement of NF-κB. Hepatocellular death is a cardinal feature of NAFLD and NASH, as characterized by the presence of swollen hepatocytes (Zhao et al., 2020). During this progress, signaling pathways related to both apoptosis and necroptosis are activated, and TNF-α acts as the original signal to launch the cascade reaction (Lu et al., 2022). Apart from the death signal of TNF-α, we also focused on the anti-inflammatory mechanism of resveratrol. For example, proinflammatory-related ILs were alleviated in experiments, suggesting the comprehensive potential of the liver-protective activity of resveratrol by inhibiting inflammation and downstream cell death. Moreover, studies have also investigated the PPAR-α signal for improvements in lipid metabolism and further confirmed that resveratrol may regulate fatty liver status via multiple targets and their crosstalk. Interestingly, the gut–liver axis also plays a role during NAFLD treatment. Intestinal tight junctions, as a gut barrier, can restrain the permeability of gut-derived endotoxins to induce TLR4/NFκB-mediated inflammation. They attenuate hepatic inflammation at least partially by improving gut barrier integrity in green tea catechins (Hodges et al., 2020). This result represents and further suggests the multidimensional regulatory role of resveratrol.

### 4.4 Challenges of resveratrol for NAFLD studies

In the present meta-analysis, we identified several issues that should be considered further. First, information on the safety of resveratrol is scarce. Although it is widely believed that resveratrol does not exhibit toxicity, its multitarget characteristic may pose a potential safety risk for other cells, which should not be ignored. Second, it is particularly important to investigate its metabolite form for clinical applications, rather than focusing on its original preparation. At present, many studies have suggested that resveratrol can be modified in the liver and intestine to generate new chemicals (Iglesias-Aguirre et al., 2022). However, studies have only proved the efficacy of resveratrol but not that of its metabolites. Therefore, there is a long way to further research in this field. Third, it is challenging to ascertain the exact progression of NAFLD. All diseases are dynamic in nature and change with time. For example, there is a close association between the development of liver injury, fibrosis, and hepatocarcinoma. The liver is rapidly repaired after damage at the early stage. However, continuous damage is a crucial factor for fibrosis accumulation and gradually toward cirrhosis. During the final stage, loss of hepatic function will ultimately lead to the development of hepatocarcinoma (Xiao et al., 2022). NAFLD development also occurs similarly. Mild NAFLD can be alleviated along with lipid elimination; however, moderate NAFLD will result in hepatocyte apoptosis and inflammation. Further several progress can considerably induce dysfunction of the microenvironment and eventually lead to the development of nonalcoholic steatohepatitis. Nevertheless, the studies included in this study did not mention the treatment stage of NAFLD. Therefore, rigorous RCTs should be designed to investigate efficacy based on disease phases.

### 4.5 Limitations

Although this study was rigorously conducted based on the PRISMA criteria, several limitations should be highlighted. 1) We could not eliminate potential heterogeneity via subgroup and sensitivity analyses. Furthermore, it was challenging to ascertain which program, including designs, modeling route, dosage, and treatment or detection time in different conditions, induced diversity. Therefore, heterogeneity may inevitably affect the conclusions and interpretations of the present study. 2) The number of clinical trials included was considerably lesser than that of preclinical experiments. This result directly suggests the shortage of translational research in resveratrol for NAFLD. Furthermore, limited clinical information constrained more precise conclusions from humans. 3) The mechanism of resveratrol was indirectly obtained from preclinical studies but not from clinical trials. Although preclinical studies can provide abundant information on how an agent functions, direct mechanistic evidence from RCTs could offer more information during application. Therefore, multiomics such as metabolomics, metagenomics, and transcriptomics should be employed for further investigations after efficacy confirmation in clinical studies. Finally, this study failed to entirely elucidate the efficacy of resveratrol on NAFLD. Therefore, we should treat the present results with caution when considering these limitations.

## 5 Conclusion

In summary, resveratrol at a dose of 50–200 mg/kg and time range of 4–8 weeks exhibits a remarkable regulatory effect on NAFLD in preclinical studies. However, the results are inconsistent in clinical trials. Considering heterogeneous factors, large-scale and single-center RCTs are warranted to investigate efficacy. Moreover, resveratrol can attenuate NAFLD mainly via liver-protective signals, including the AMPK/Sirt1 and anti-inflammatory signaling pathways.

## Data Availability

The original contributions presented in the study are included in the article/Supplementary Material, further inquiries can be directed to the corresponding author.

## Supplementary material

SUPPLEMENTARY FIGURE S1 Risk of bias summary assessment for preclinical studies.

SUPPLEMENTARY FIGURE S2 Methodological quality assessment of clinical trials.

SUPPLEMENTARY FIGURE S3 Effect of resveratrol on LDL-C and HDL-C level in preclinical studies.

SUPPLEMENTARY FIGURE S4 Effect of resveratrol on serum insulin level in preclinical studies.

SUPPLEMENTARY FIGURE S5 Effect of resveratrol on MDA and SOD level in preclinical studies.

SUPPLEMENTARY FIGURE S6 Effect of resveratrol on MDA and SOD level in preclinical studies.

SUPPLEMENTARY FIGURE S7 Effect of resveratrol on MDA and SOD level in preclinical studies.

SUPPLEMENTARY FIGURE S8 Effect of resveratrol on TNF-α and IL-6 level in preclinical studies.

SUPPLEMENTARY FIGURE S9 Effect of resveratrol on IL-1β level in preclinical studies.

SUPPLEMENTARY FIGURE S10 Subgroup analysis of TG based on animal species.

SUPPLEMENTARY FIGURE S11 Subgroup analysis of TG based on dosage.

SUPPLEMENTARY FIGURE S12 Subgroup analysis of ALT based on animal species.

SUPPLEMENTARY FIGURE S13 Subgroup analysis of ALT based on dosage.

SUPPLEMENTARY FIGURE S14 Effect of resveratrol on body weight and BMI in clinical trials.

SUPPLEMENTARY FIGURE S15 Effect of resveratrol onWC andWHR in clinical trials.

SUPPLEMENTARY FIGURE S16 Effect of resveratrol on LDL-C and HDL-C level in clinical trials.

SUPPLEMENTARY FIGURE S17 Effect of resveratrol on insulin and HOMA-IR level in clinical trials.

SUPPLEMENTARY FIGURE S18 Effect of resveratrol on glucose level in clinical trials.

SUPPLEMENTARY FIGURE S19 Effect of resveratrol on TNF-α in clinical trials.

SUPPLEMENTARY FIGURE S20 Egger’s test for ALT and TG in preclinical studies.

SUPPLEMENTARY TABLE S1 Search strategy in PubMed.

SUPPLEMENTARY TABLE S2 Characteristics of preclinical studies.

SUPPLEMENTARY TABLE S3 Characteristics of clinical trials.

SUPPLEMENTARY TABLE S4 Possible mechanism of resveratrol on NAFLD.

## Abbreviations

AMPK, adenosine 5′-monophosphate (AMP)-activated protein kinase; BMI, body mass index; BW, body weight; CI, confidence intervals; EBM, evidence-based medicine; HOMA-IR, homeostasis model assessment of insulin resistance; IL-1β, interleukin-1β; IL-6, interleukin-6; MCD, methionine-choline-deficient diet; NAFLD, nonalcoholic fatty liver disease; PRISMA, the Preferred Reporting Items for Systematic Reviews and Meta-Analyses; RCTs, randomized clinical trials; ROS, reactive oxygen species; SD, standard deviation; SMD, standardized mean difference; SOD, superoxide dismutase; TNF-α, tumor necrosis factor-α; WC, waist circumference; WHR, waist-to-hip ratio.
