# Supplementary figures and images for: Integrative evidence construction for resveratrol treatment of nonalcoholic fatty liver disease: preclinical and clinical meta-analyses
Source: Front Pharmacol. 2023 Sep 12;14:1230783. doi: 10.3389/fphar.2023.1230783 (PMC10520779; doi:10.3389/fphar.2023.1230783)

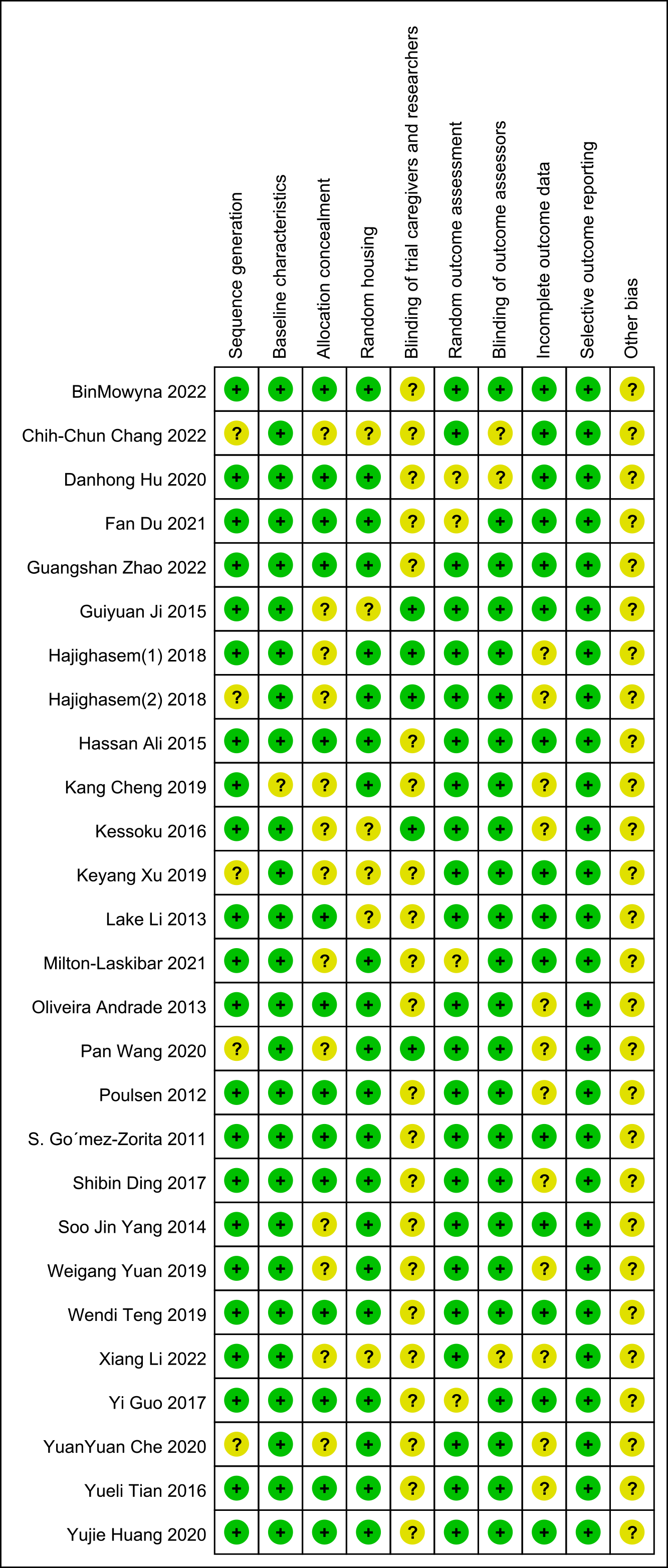

Supplement: Supplementary file 1 [file DataSheet1.ZIP › Supplementary Figures/Supplementary Figure 1.tif]

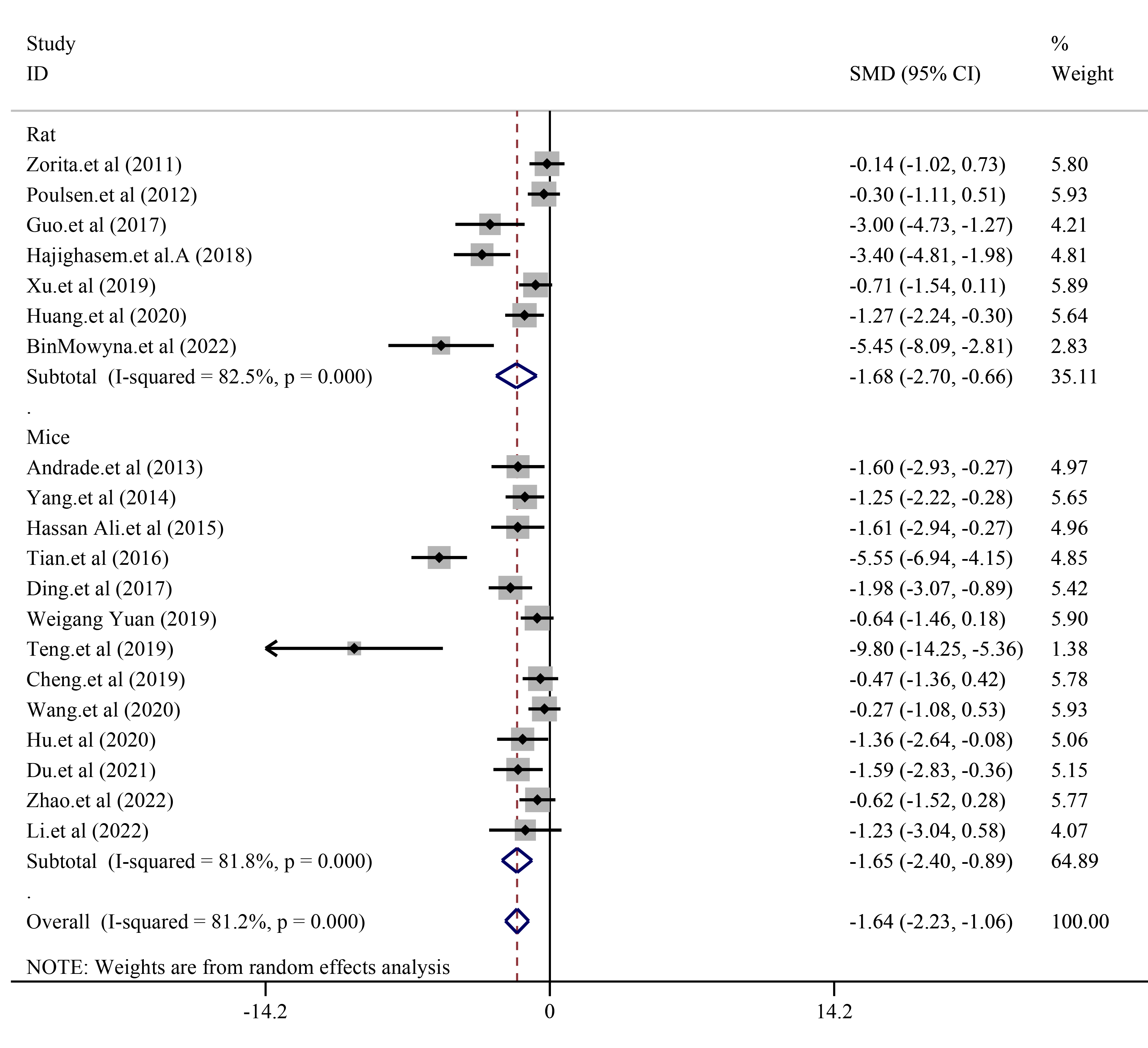

Supplement: Supplementary file 1 [file DataSheet1.ZIP › Supplementary Figures/Supplementary Figure 10.tif]

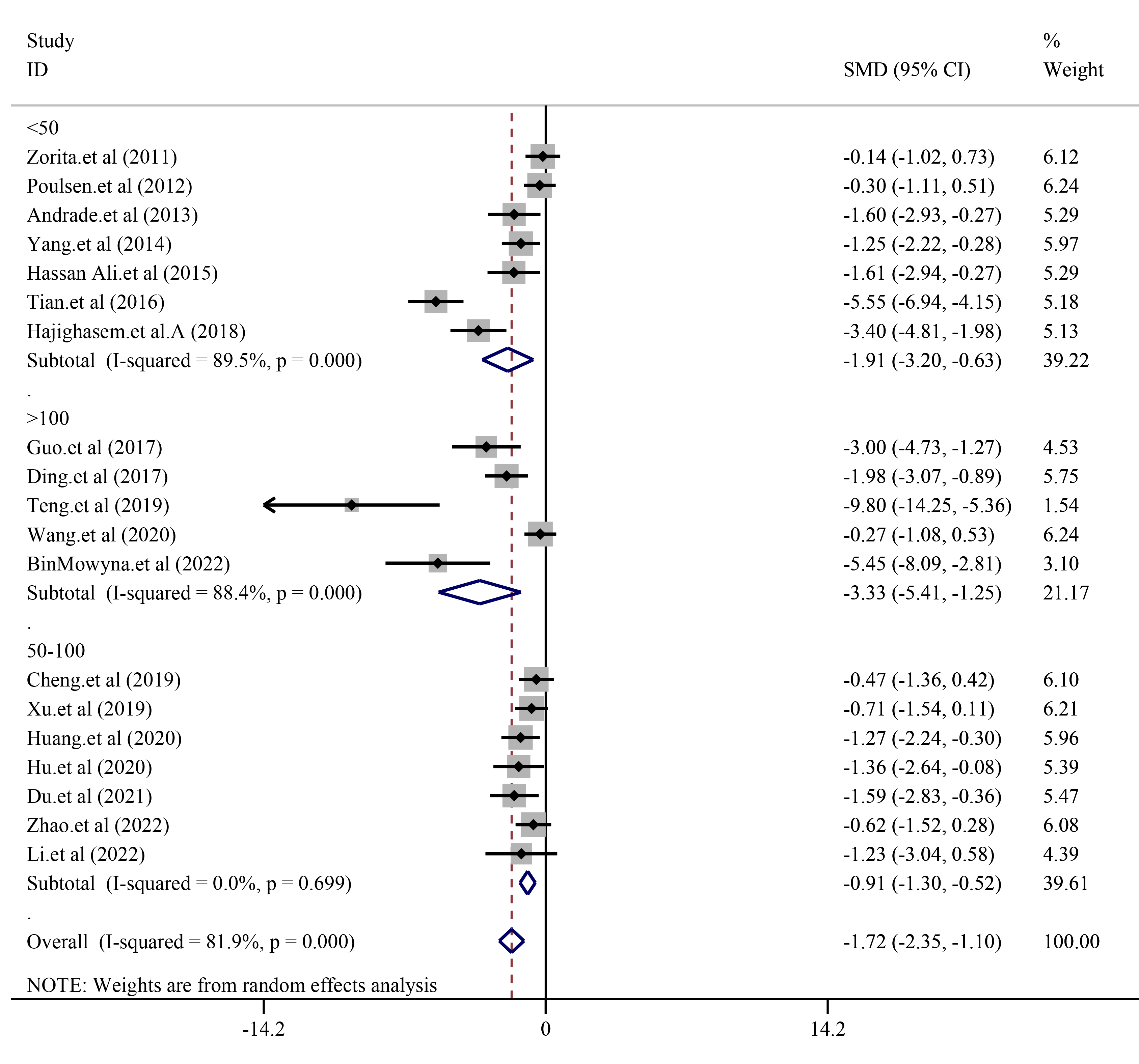

Supplement: Supplementary file 1 [file DataSheet1.ZIP › Supplementary Figures/Supplementary Figure 11.tif]

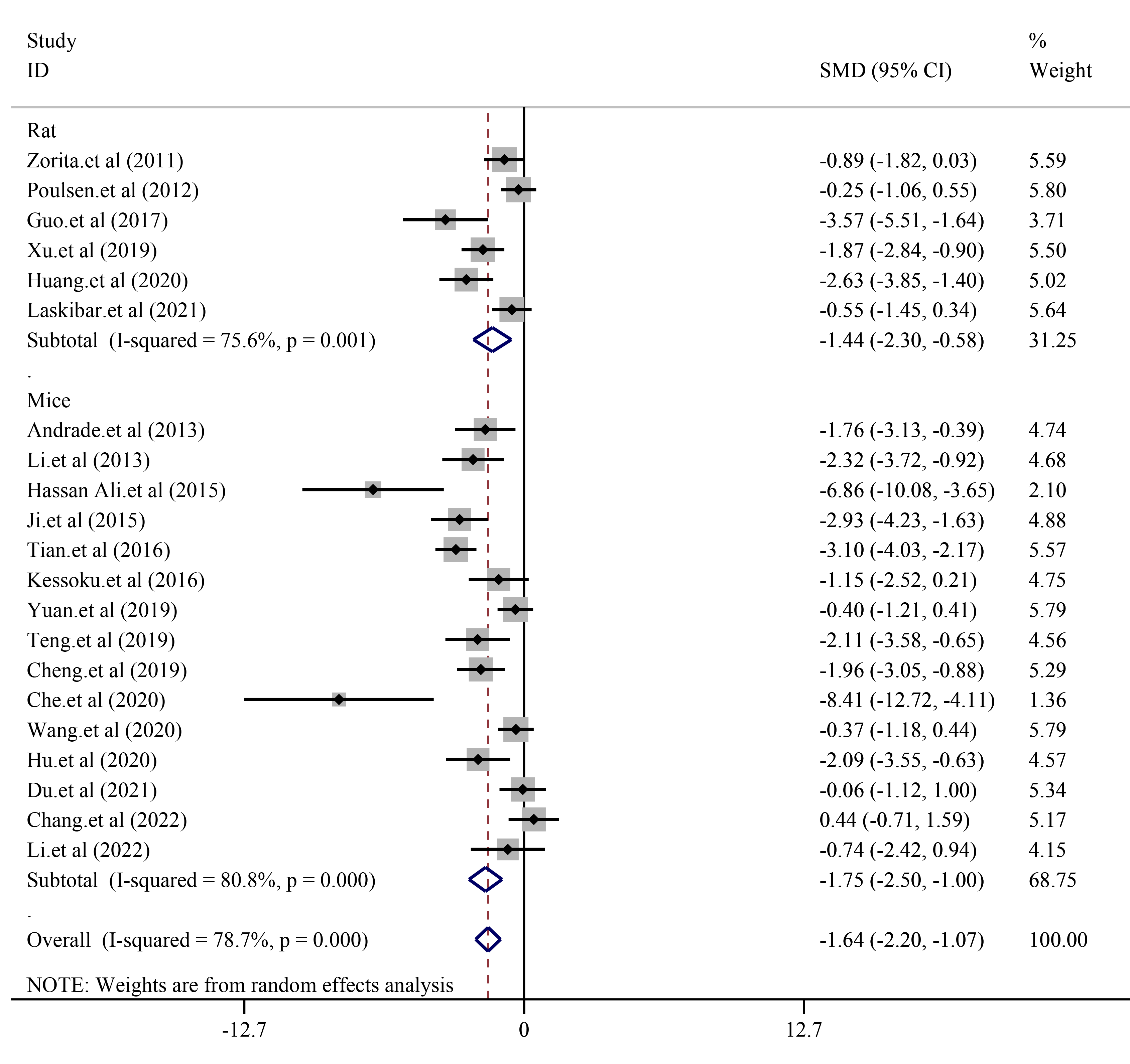

Supplement: Supplementary file 1 [file DataSheet1.ZIP › Supplementary Figures/Supplementary Figure 12.tif]

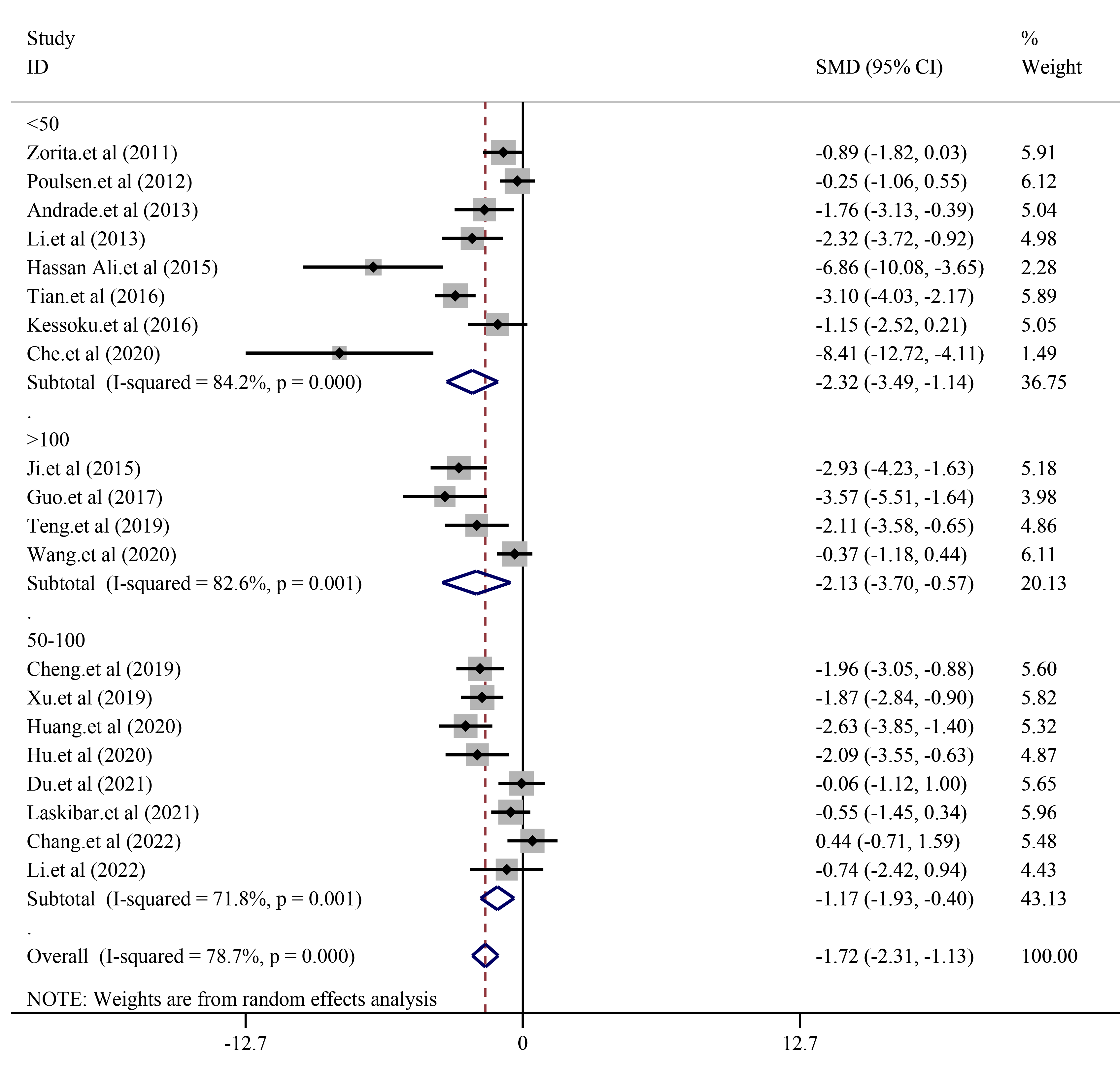

Supplement: Supplementary file 1 [file DataSheet1.ZIP › Supplementary Figures/Supplementary Figure 13.tif]

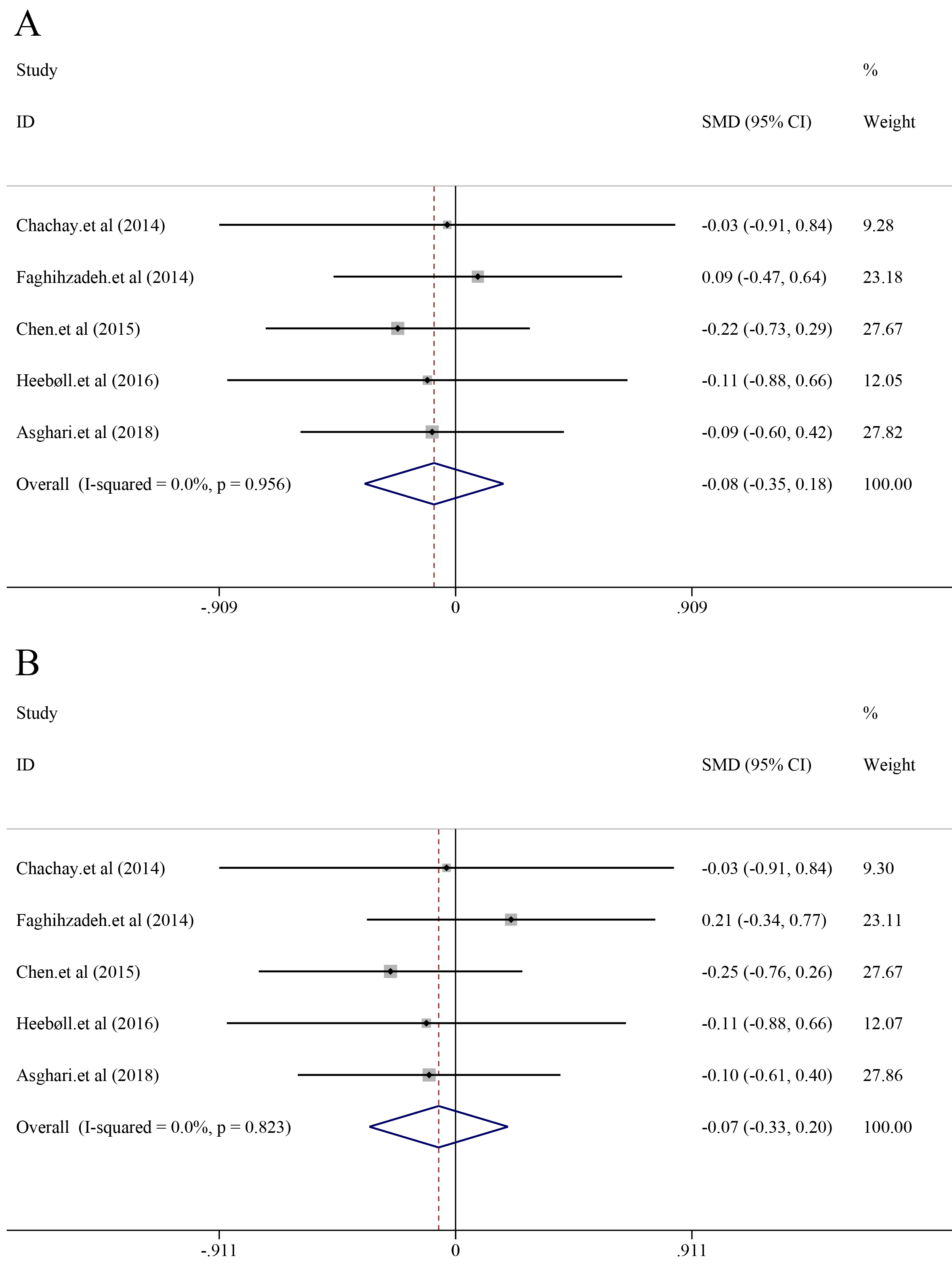

Supplement: Supplementary file 1 [file DataSheet1.ZIP › Supplementary Figures/Supplementary Figure 14.tif]

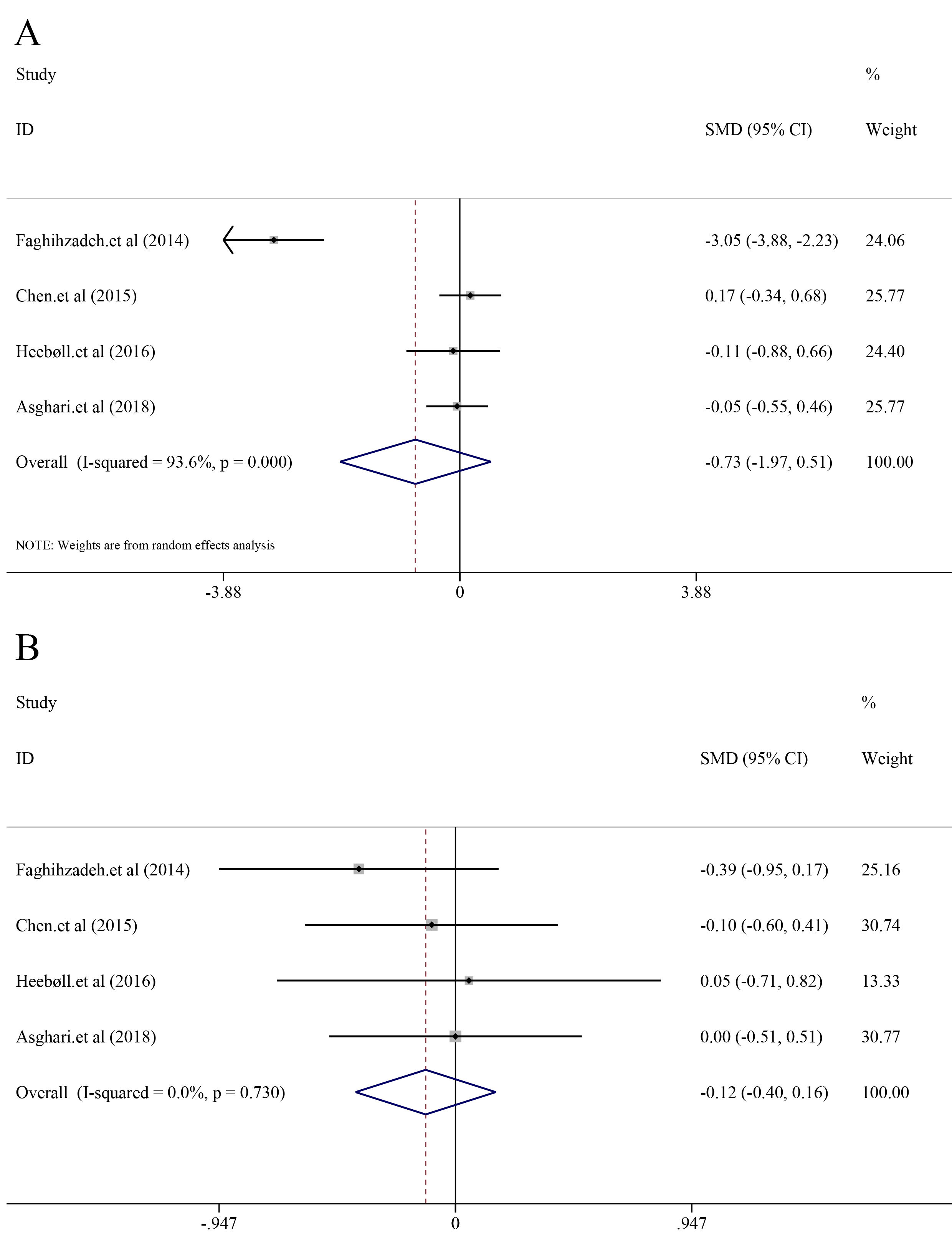

Supplement: Supplementary file 1 [file DataSheet1.ZIP › Supplementary Figures/Supplementary Figure 15.tif]

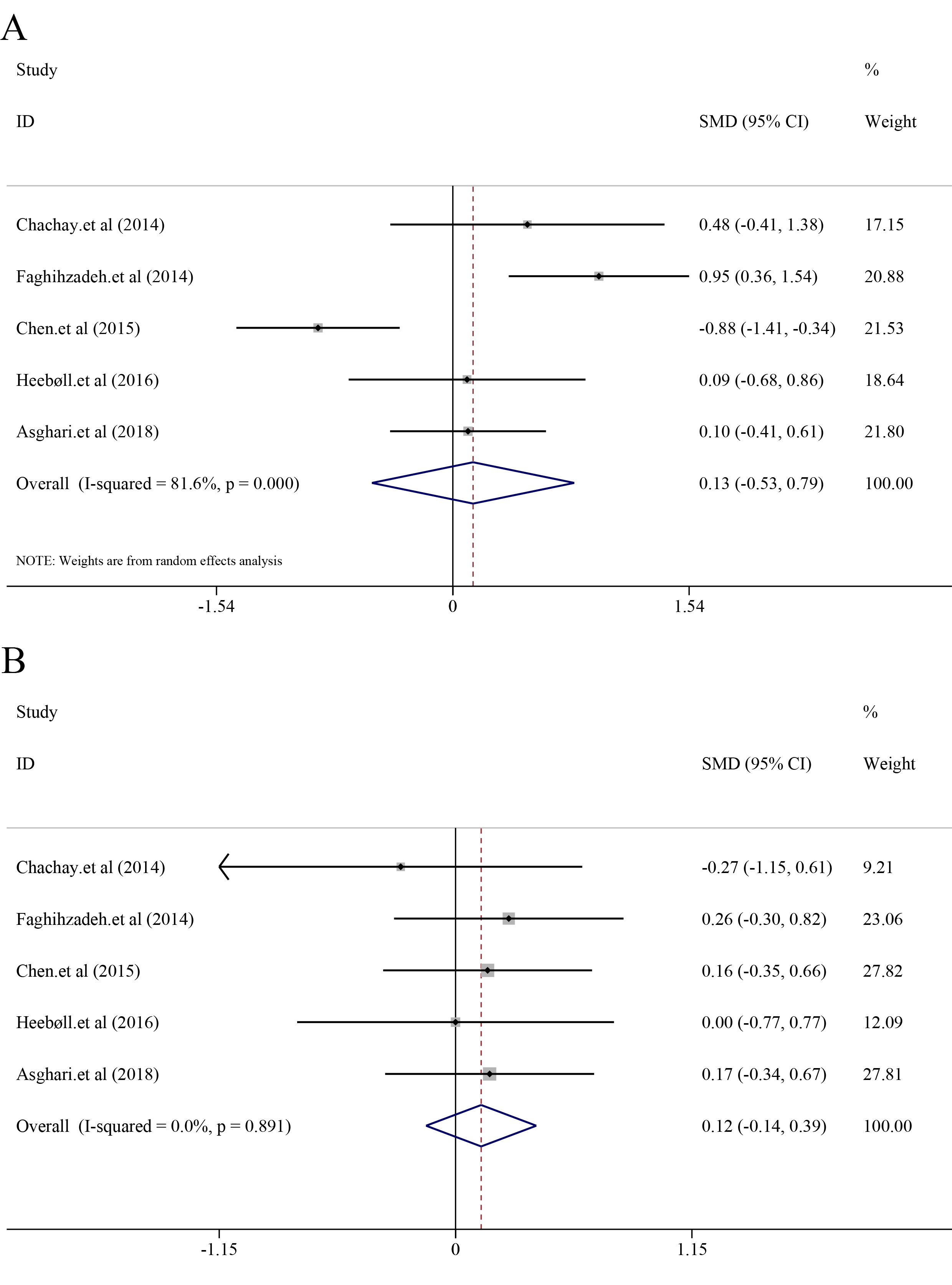

Supplement: Supplementary file 1 [file DataSheet1.ZIP › Supplementary Figures/Supplementary Figure 16.tif]

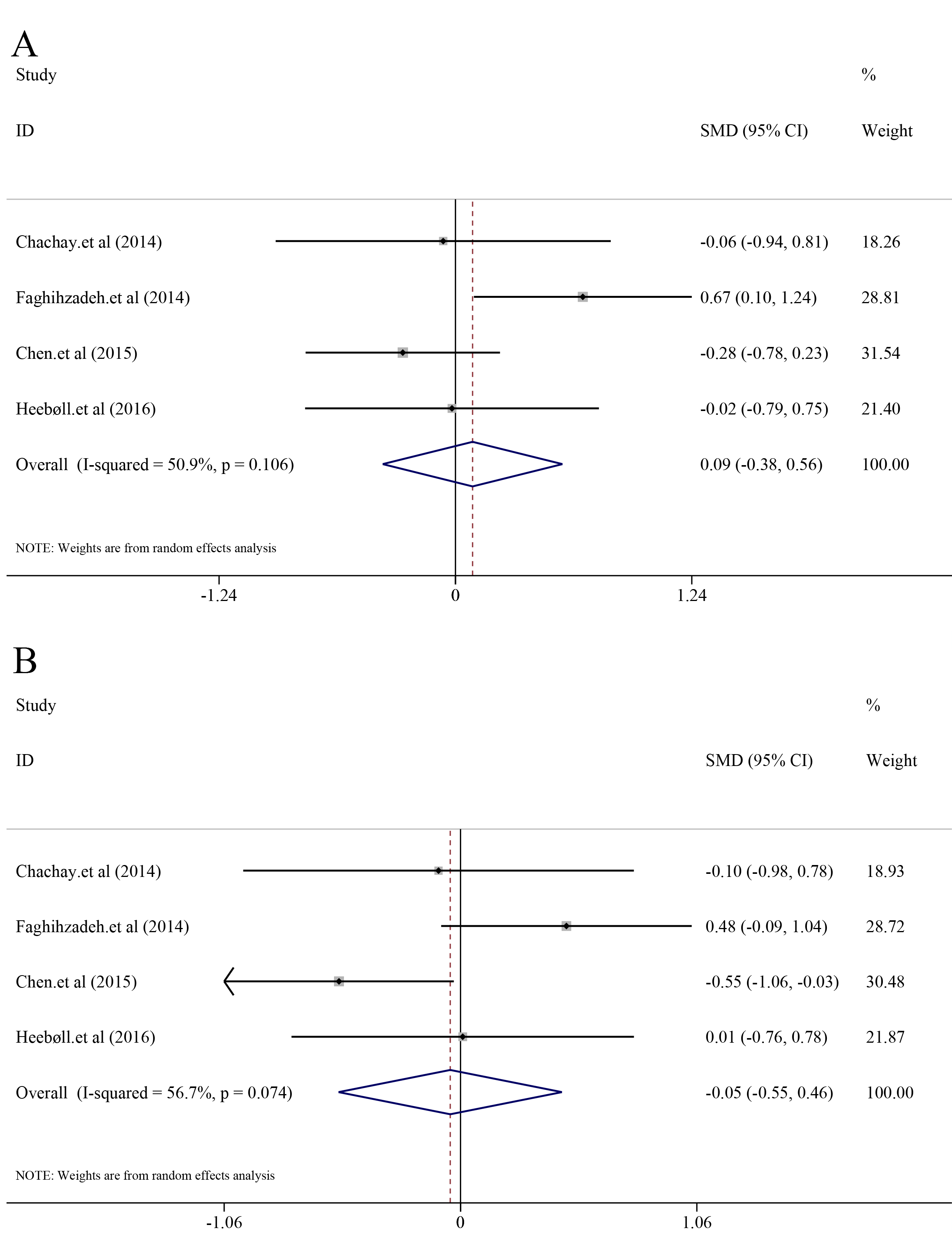

Supplement: Supplementary file 1 [file DataSheet1.ZIP › Supplementary Figures/Supplementary Figure 17.tif]

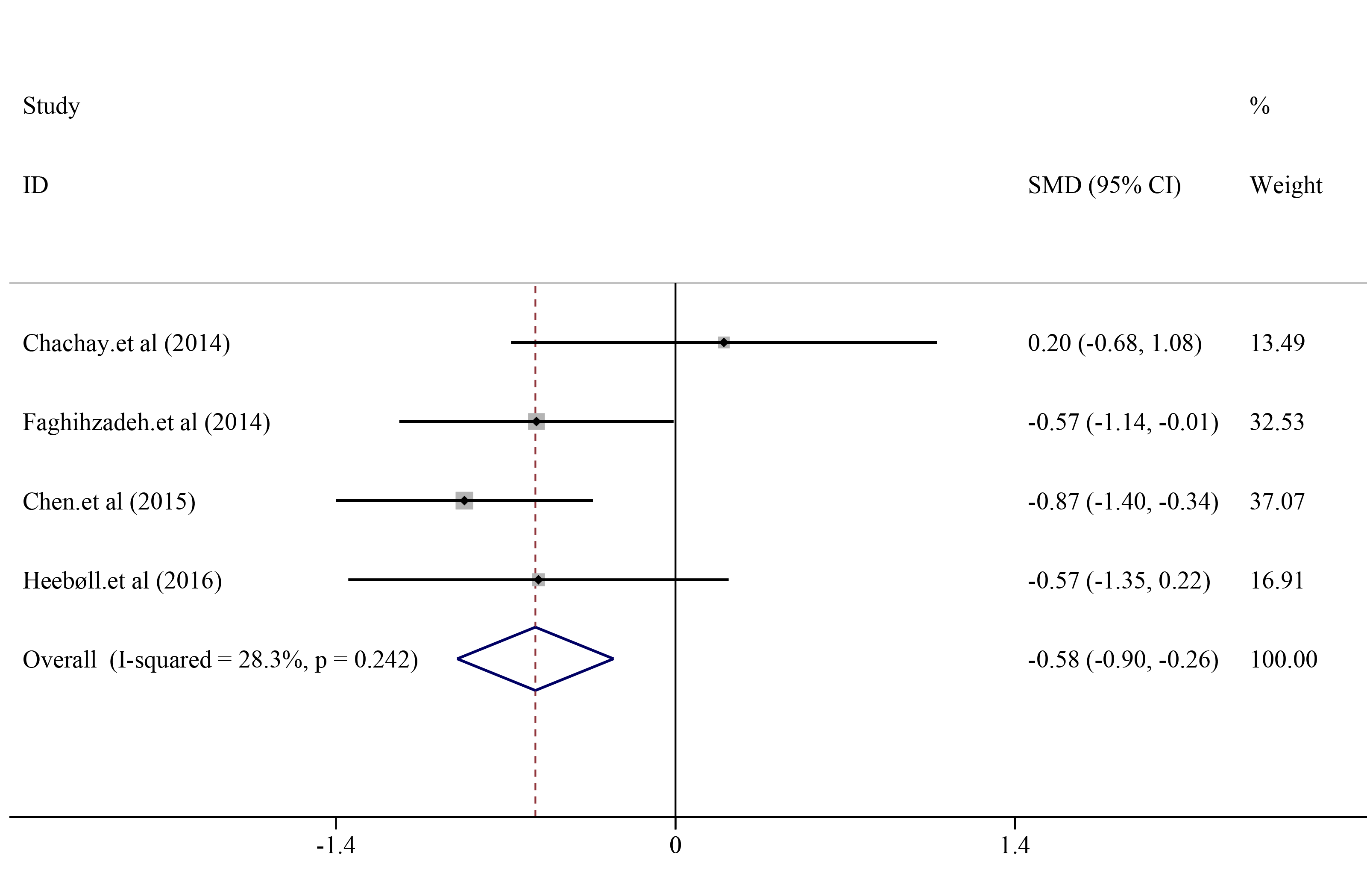

Supplement: Supplementary file 1 [file DataSheet1.ZIP › Supplementary Figures/Supplementary Figure 18.tif]

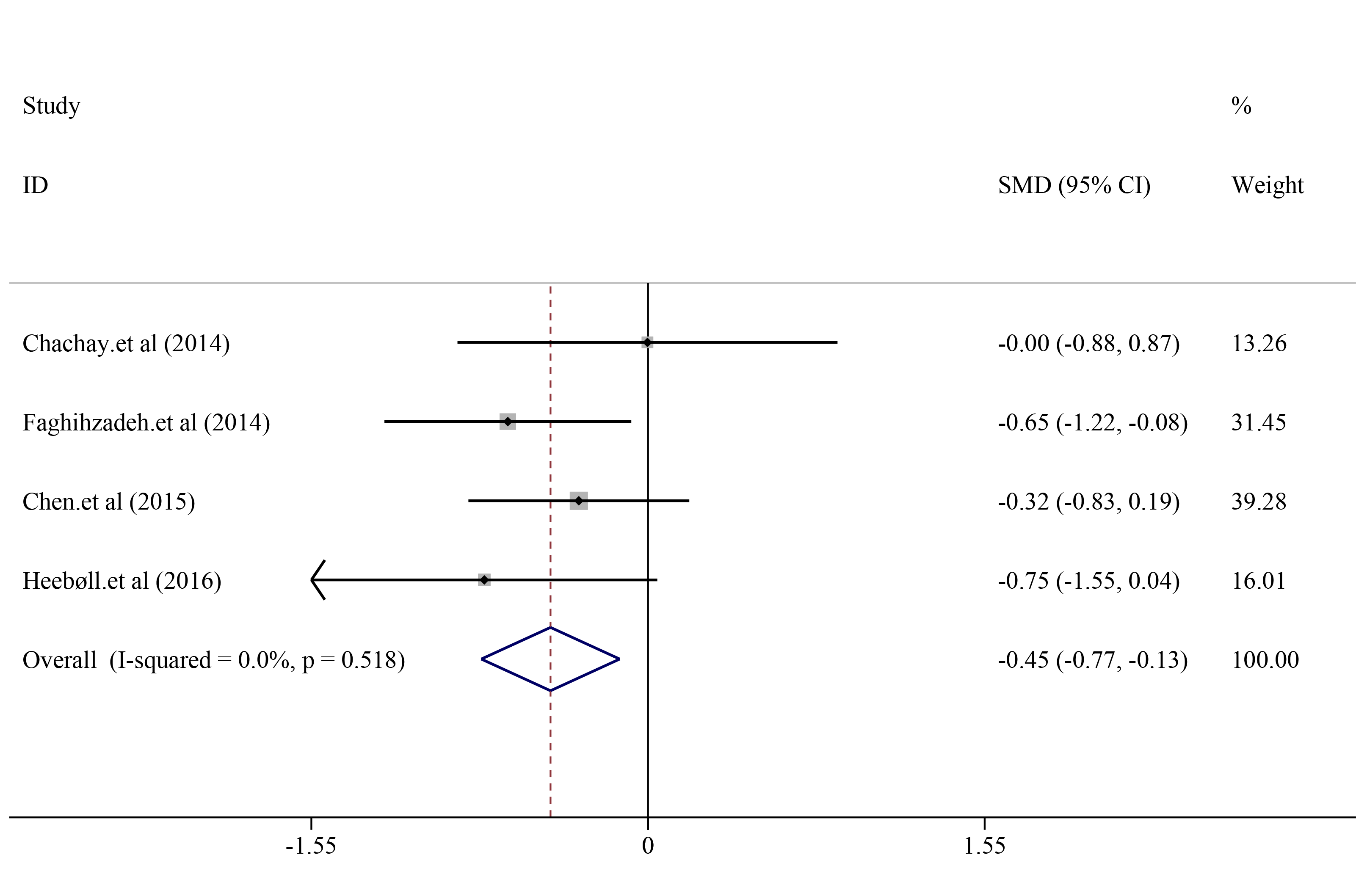

Supplement: Supplementary file 1 [file DataSheet1.ZIP › Supplementary Figures/Supplementary Figure 19.tif]

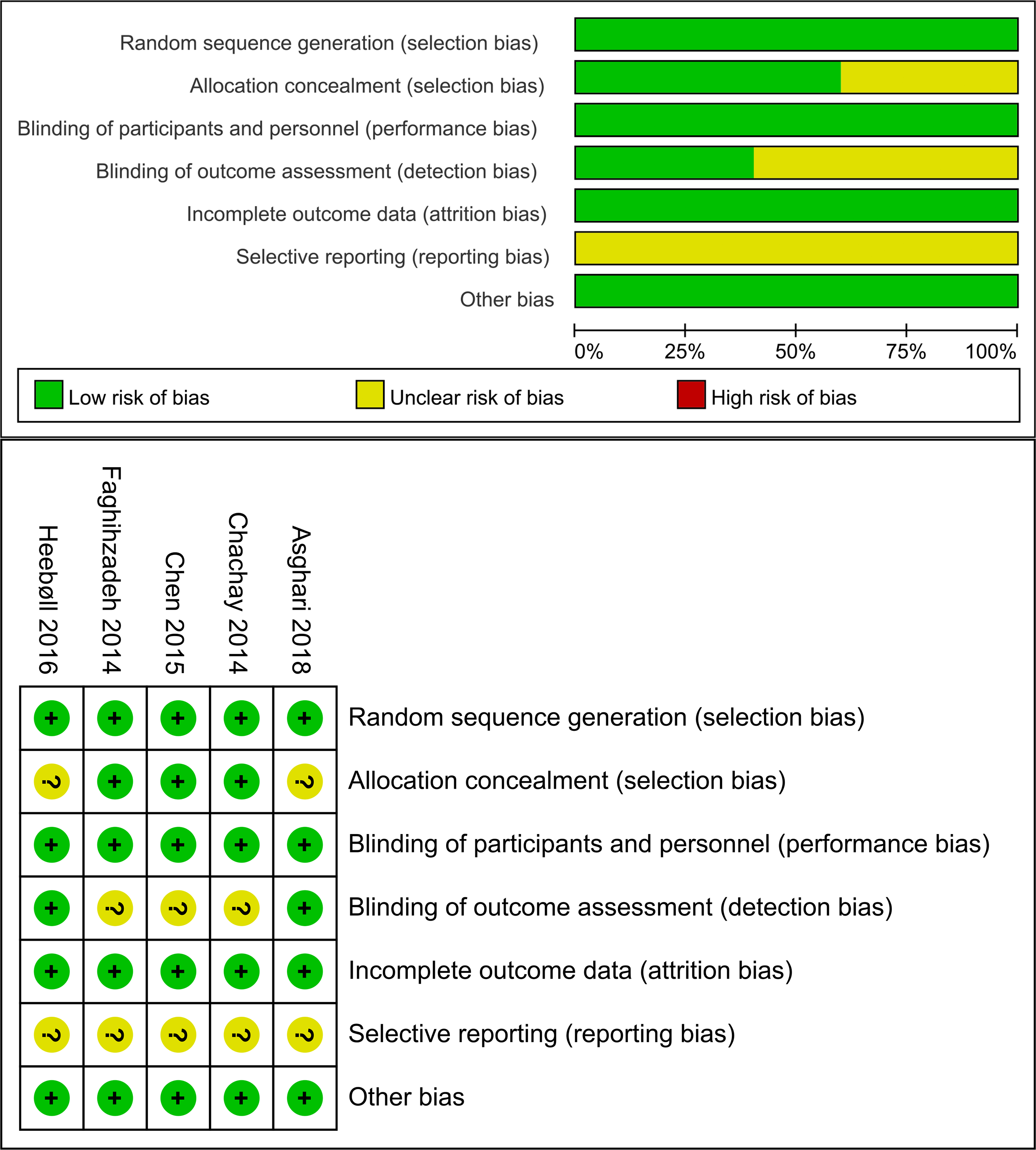

Supplement: Supplementary file 1 [file DataSheet1.ZIP › Supplementary Figures/Supplementary Figure 2.tif]

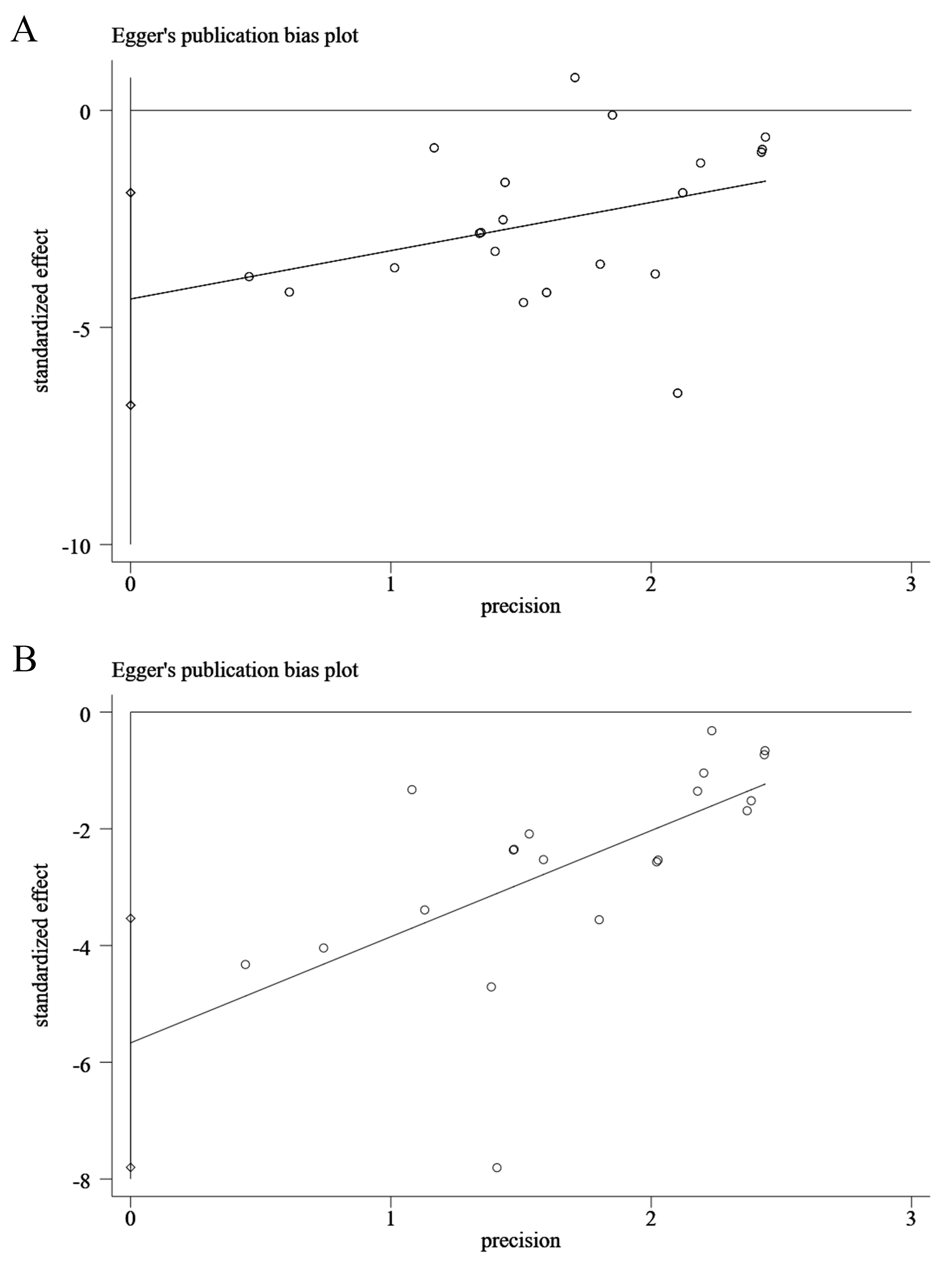

Supplement: Supplementary file 1 [file DataSheet1.ZIP › Supplementary Figures/Supplementary Figure 20.tif]

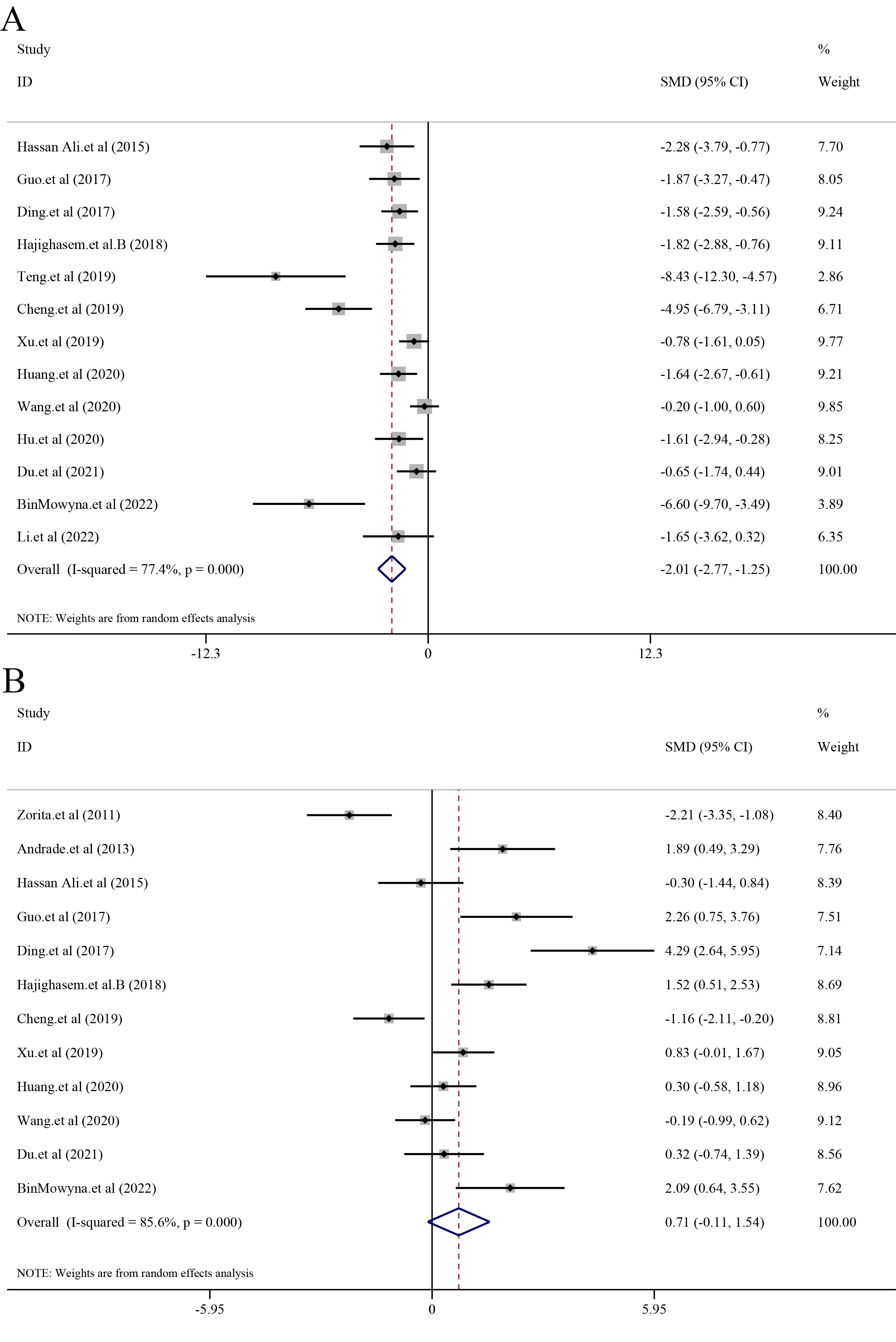

Supplement: Supplementary file 1 [file DataSheet1.ZIP › Supplementary Figures/Supplementary Figure 3.tif]

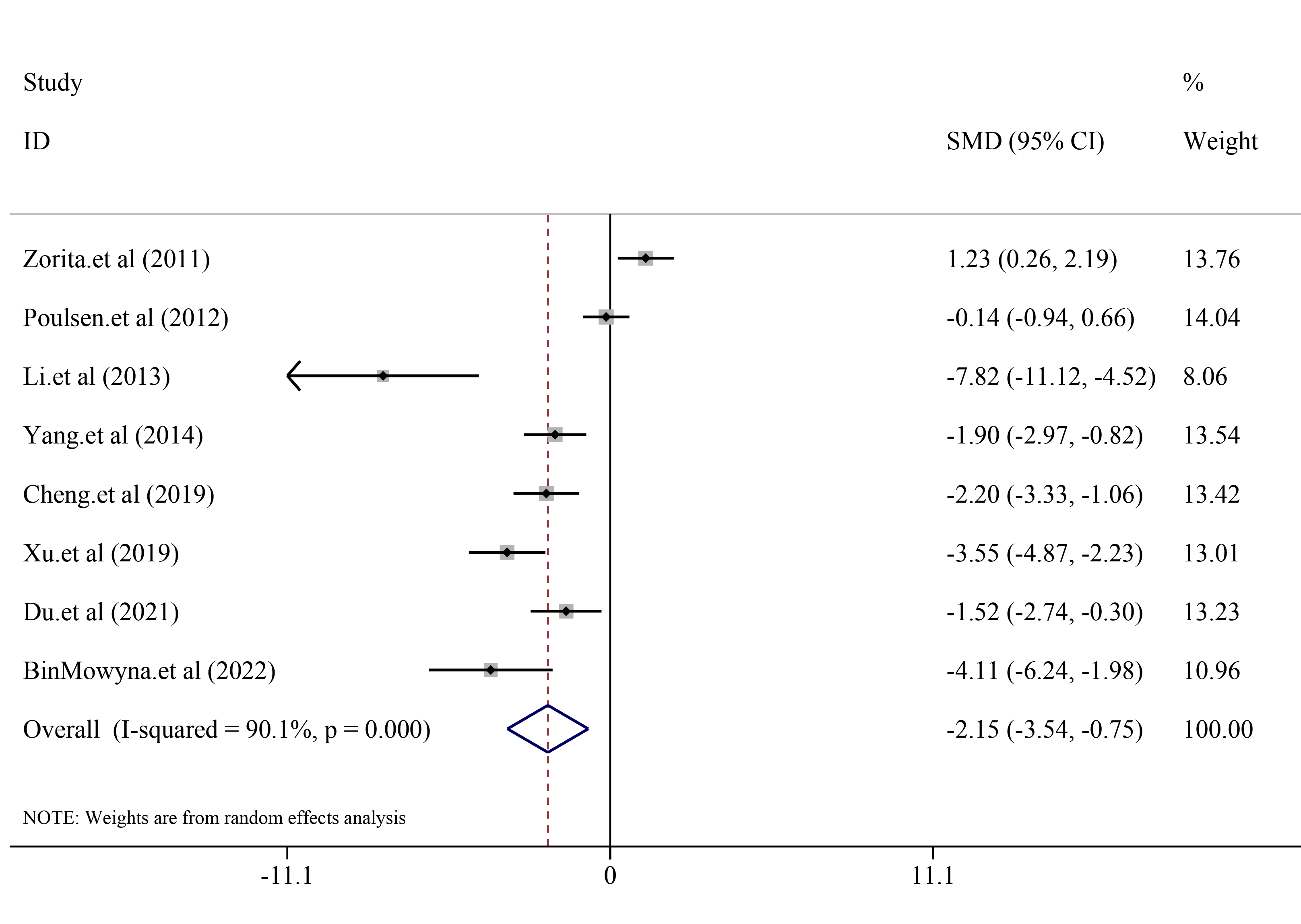

Supplement: Supplementary file 1 [file DataSheet1.ZIP › Supplementary Figures/Supplementary Figure 4.tif]

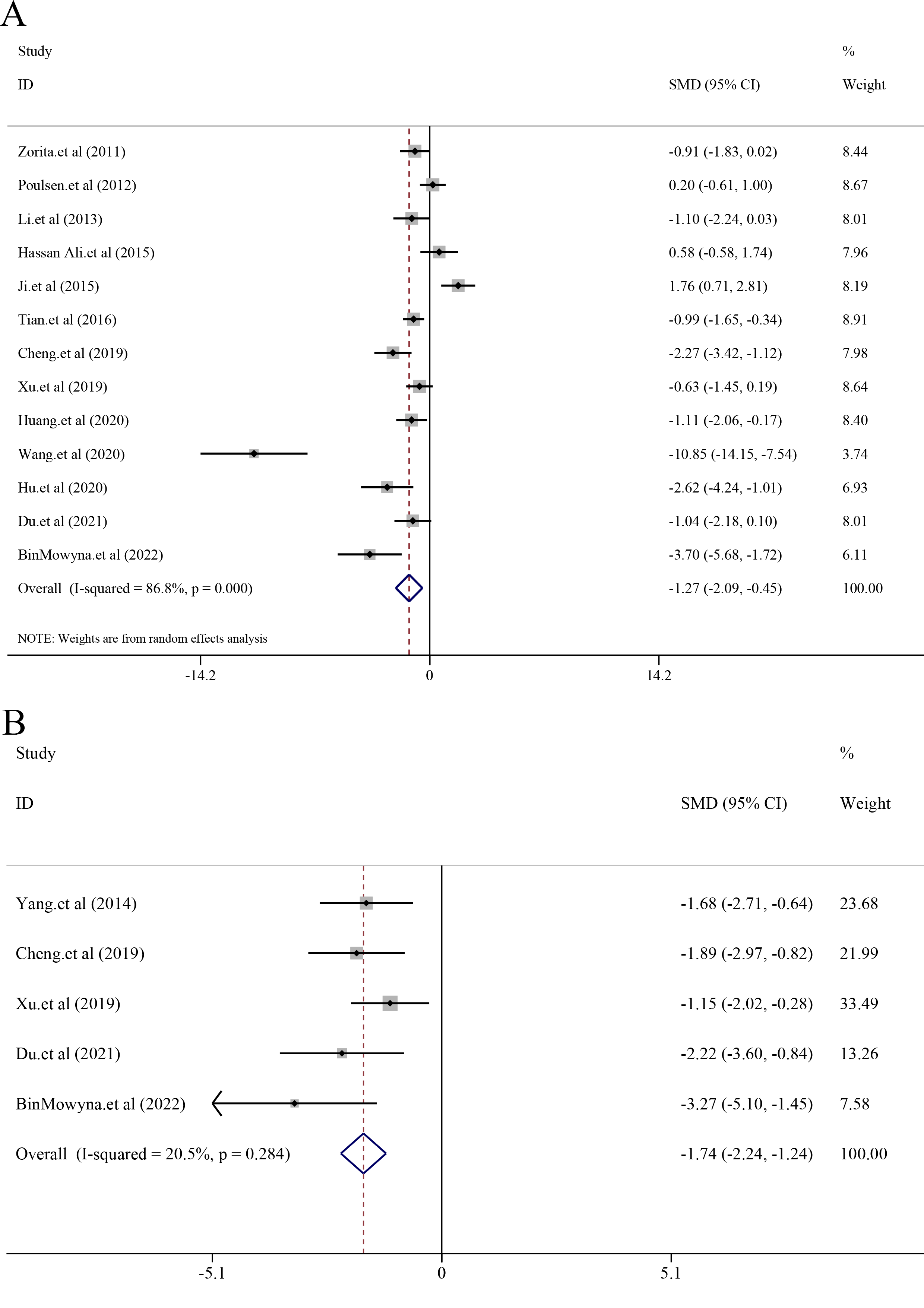

Supplement: Supplementary file 1 [file DataSheet1.ZIP › Supplementary Figures/Supplementary Figure 5.tif]

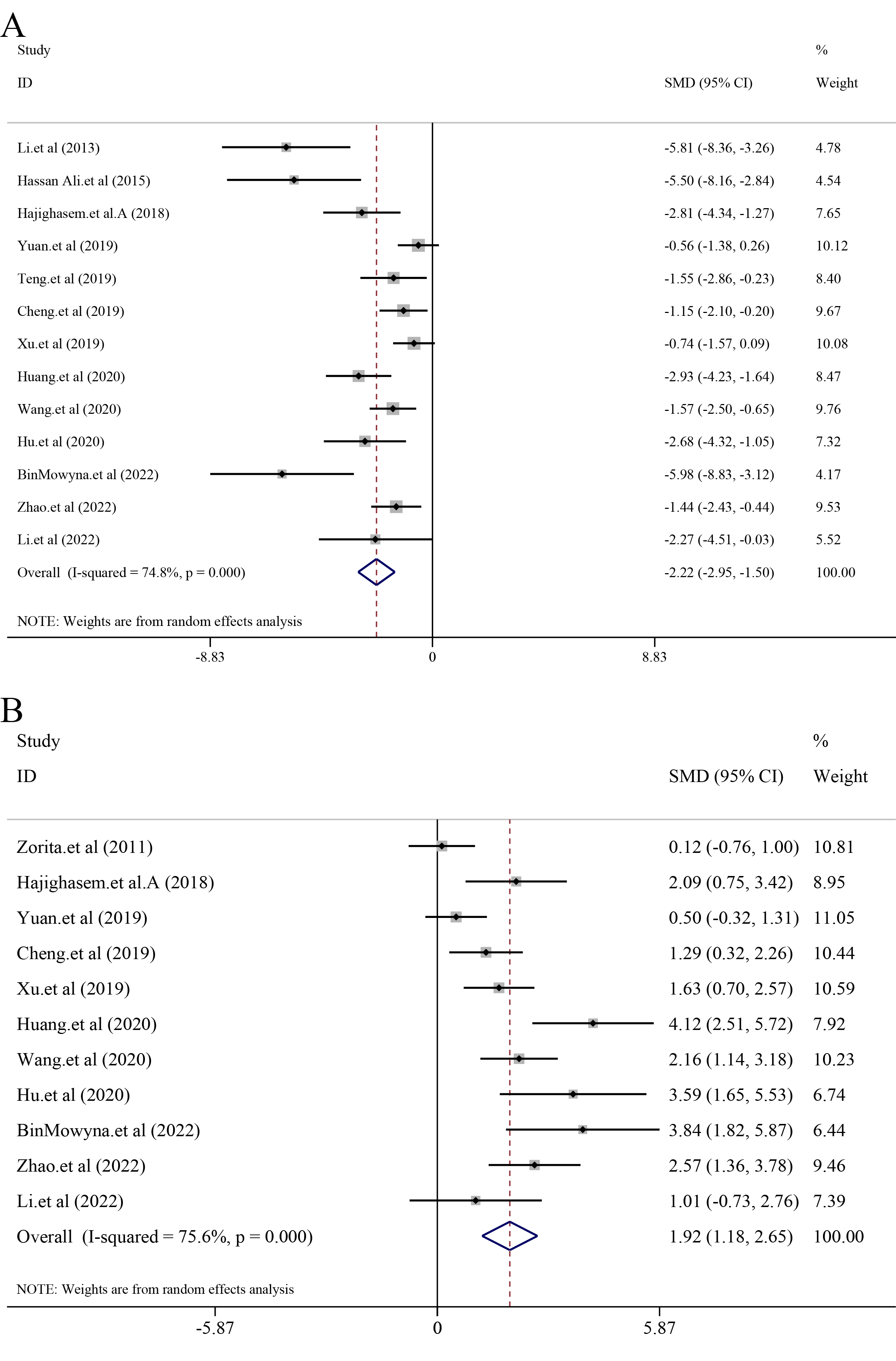

Supplement: Supplementary file 1 [file DataSheet1.ZIP › Supplementary Figures/Supplementary Figure 6.tif]

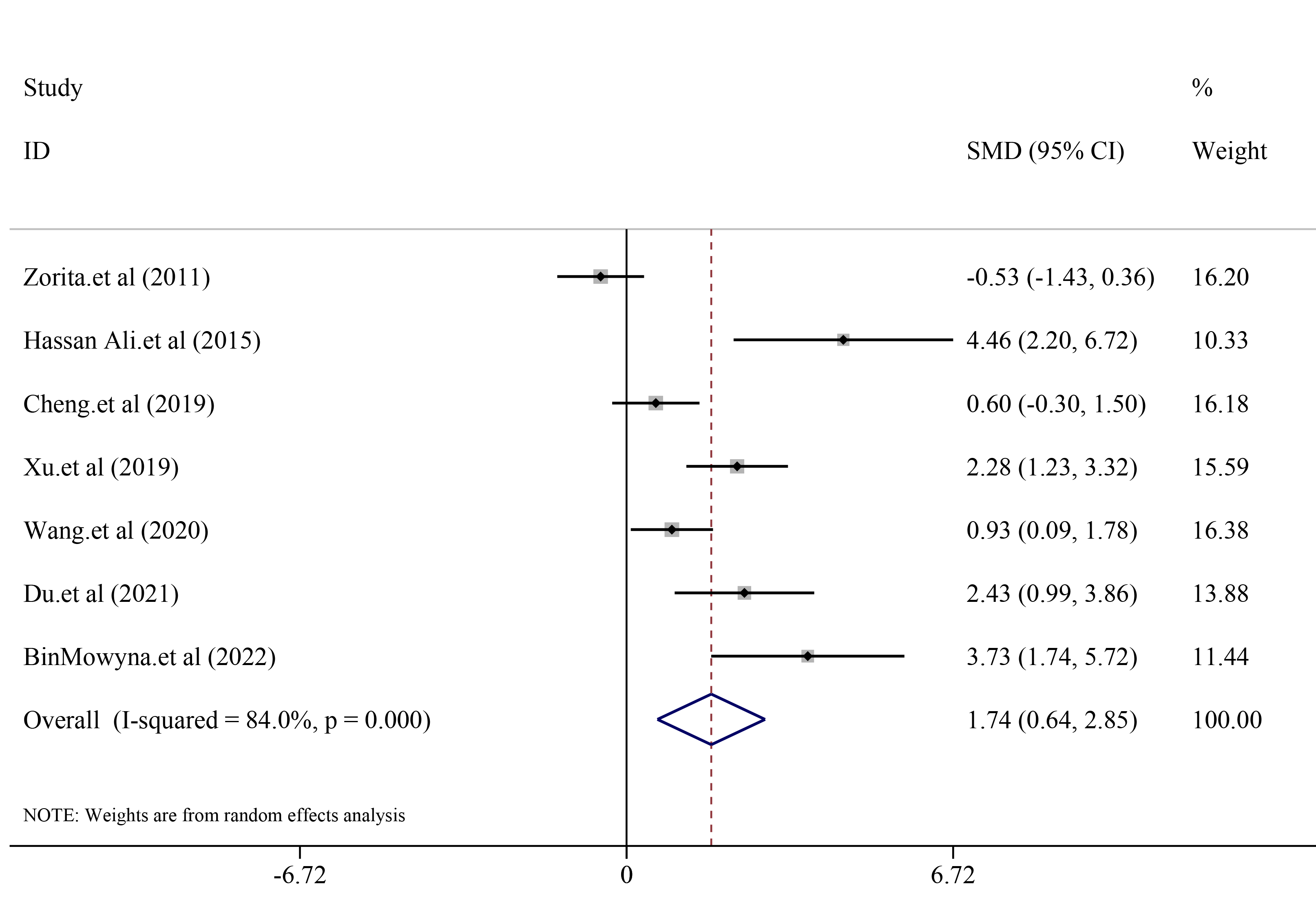

Supplement: Supplementary file 1 [file DataSheet1.ZIP › Supplementary Figures/Supplementary Figure 7.tif]

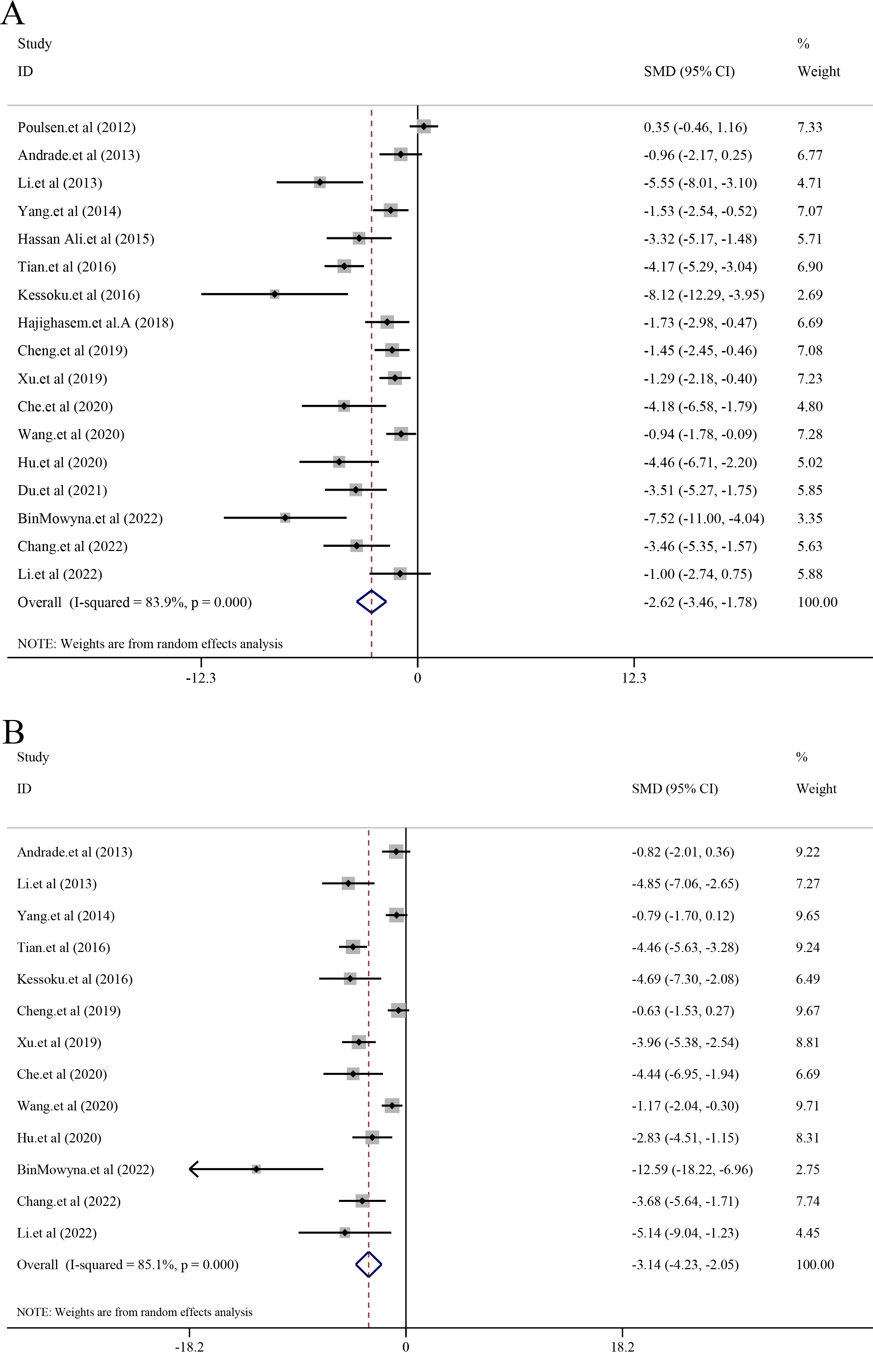

Supplement: Supplementary file 1 [file DataSheet1.ZIP › Supplementary Figures/Supplementary Figure 8.tif]

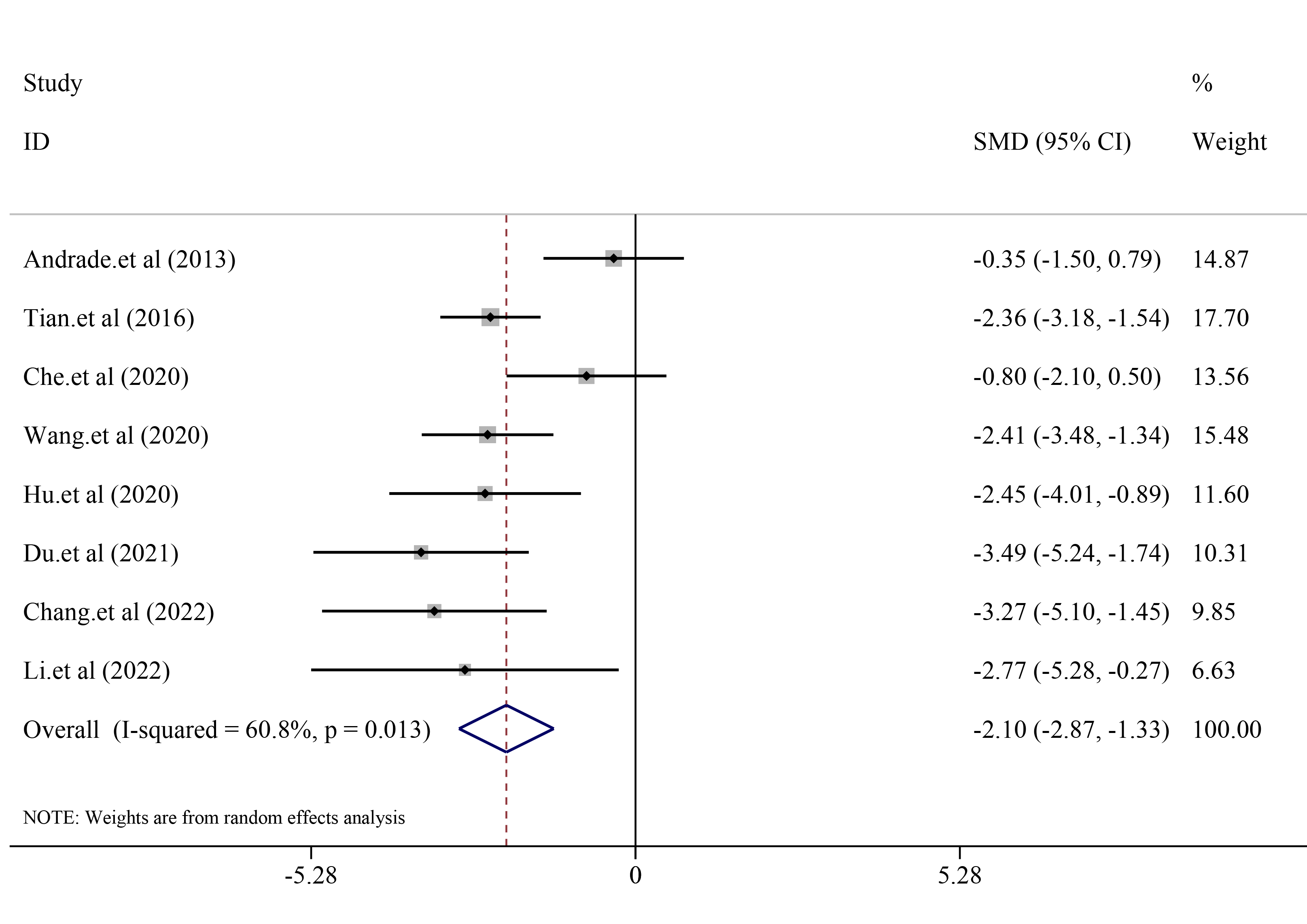

Supplement: Supplementary file 1 [file DataSheet1.ZIP › Supplementary Figures/Supplementary Figure 9.tif]
